# E2F4 regulates transcriptional activation in mouse embryonic stem cells independently of the RB family

**DOI:** 10.1038/s41467-019-10901-x

**Published:** 2019-07-03

**Authors:** Jenny Hsu, Julia Arand, Andrea Chaikovsky, Nancie A. Mooney, Janos Demeter, Caileen M. Brison, Romane Oliverio, Hannes Vogel, Seth M. Rubin, Peter K. Jackson, Julien Sage

**Affiliations:** 1 Department of Pediatrics, 300 Pasteur Drive, Stanford University, Stanford, CA 94305 USA; 2 Department of Genetics, 300 Pasteur Drive, Stanford University, Stanford, CA 94305 USA; 3 Baxter Laboratory, Department of Microbiology & Immunology, 300 Pasteur Drive, Stanford University, Stanford, CA 94305 USA; 4 Department of Chemistry and Biochemistry, University of California, 1156 High Street, Santa Cruz, CA 95064 USA; 5 Department of Pathology, 300 Pasteur Drive, Stanford University, Stanford, CA 94305 USA

**Keywords:** Cell division, Gene regulation, Cell division, Transcription, Embryonic stem cells

## Abstract

E2F transcription factors are central regulators of cell division and cell fate decisions. E2F4 often represents the predominant E2F activity in cells. E2F4 is a transcriptional repressor implicated in cell cycle arrest and whose repressive activity depends on its interaction with members of the RB family. Here we show that E2F4 is important for the proliferation and the survival of mouse embryonic stem cells. In these cells, E2F4 acts in part as a transcriptional activator that promotes the expression of cell cycle genes. This role for E2F4 is independent of the RB family. Furthermore, E2F4 functionally interacts with chromatin regulators associated with gene activation and we observed decreased histone acetylation at the promoters of cell cycle genes and E2F targets upon loss of E2F4 in RB family-mutant cells. Taken together, our findings uncover a non-canonical role for E2F4 that provide insights into the biology of rapidly dividing cells.

## Abstract (web summary)

E2F transcription factors are regulators of cell division and cell fate decisions. Here the authors show that E2F4 is important for proliferation and survival of mouse ESCs, independent of the RB family, and that E2F4 interacts with chromatin regulators associated with gene activation.

## Introduction

Regulation of the cell cycle is a basic biological process that ensures the function and health of the entire organism. The proliferation of stem/precursor cells, the terminal differentiation of post-mitotic cells, and the establishment and maintenance of quiescent and senescent cells all require proper cell cycle regulation and are all critical for proper tissue development and homeostasis (reviewed in^1^). Aberrant cell cycle progression is linked to tumorigenesis, and blocks in cell cycle entry are linked to aging and age-related diseases (reviewed in^2,3^). Thus, a better understanding of cell cycle principles, including in stem cells, remains an important aspect of understanding human biology and improving human health.

The RB/E2F module is a well-established regulator of the G1/S transition of the cell cycle. It consists, in part, of RB and its family members p107 and p130, and the E2F family of transcription factors. The “classical” E2Fs (E2F1, E2F2, E2F3a, E2F3b, E2F4, and E2F5) can physically associate with the RB family proteins, which regulate their activity (reviewed in^4–6^). These E2Fs can be further subdivided into canonical “repressors” (E2F3b, E2F4, E2F5) and “activators” (E2F1, E2F2, E2F3a) depending on their purported activity at the promoters of cell cycle genes. While “repressor” E2Fs form complexes with the RB family proteins to inhibit the expression of cell cycle genes at the G1/S transition, “activator” E2Fs upregulate the expression of these genes to promote cell cycle entry. Binding to RB inhibits the transactivational potential of the activator E2Fs. Thus, this interplay between the “repressor” and “activator” E2Fs mediated by their association with the RB family proteins, is thought to ensure proper cell cycle progression^4^. A vast majority of human tumors carry alterations that inactivate the function of the RB family, and therefore characterizing the effects of this inactivation is of central biological importance. However, we still do not fully understand the molecular and cellular roles of the module components, including their interactions with each other and with context-dependent cofactors (reviewed in^7^).

The most ubiquitously and strongly expressed E2F family member, E2F4, has long been classified as a “repressor” E2F that silences the expression of cell cycle genes during G0 and G1 in conjunction with the RB family proteins (reviewed in^8^). E2F4 is thought to rely on the RB family proteins for translocation into the nucleus in G0/G1 as well as for repression of cell cycle genes^9^. During S phase, hyperphosphorylation of the RB family proteins prevents their binding to E2F4, and E2F4 is exported to the cytoplasm^10^, allowing activator E2Fs, which possess a nuclear localization signal, to promote the transcription of cell cycle genes. Although E2F4 contains a transactivation domain similar to that of activator E2Fs, binding to RB family members blocks this domain. The co-incidence of E2F4 release from RB family members and its export from the nucleus supports a model in which  E2F4 does not act as a transcriptional activator^11,12^.

Despite this well-established model, evidence from overexpression studies and in specific cell types such as fast-cycling intestinal cells suggests that E2F4 has the ability to activate genes and/or the proliferation of cells in some contexts (e.g.^13–17^). In multiciliated cells, E2F4 and its close homolog E2F5 can promote the expression of genes involved in centriole assembly and ciliogenesis^18–20^. However, these observations and other recent studies^21,22^ often rely on correlative analyses in cells with loss of E2F4 function or E2F4 overexpression. For instance, *E2f4* knockout mice exhibit a reduced or absent crypt region and poorly developed villi in the intestines, which may suggest a pro-proliferative role for E2F4^23^ but might also be due to developmental defects and general poor health^24^. Overall, whether E2F4 can function as a transcriptional activator in physiological contexts remains largely unexplored.

Mouse embryonic stem cells (mESCs) display little to no G1 phase but express surprisingly high levels of E2F4 (reviewed in^25^). This observation led us to examine the consequences of E2F4 loss in these cells. We found that E2F4 normally promotes the expansion of mESCs in part by directly activating the transcription of cell cycle genes. We also found that this role for E2F4 is independent of the RB family and may rely on the activity of histone acetyltransferases. These data provide conclusive evidence that E2F4 can function as a transcriptional activator in a biologically relevant context.

## Results

### E2F4 is highly expressed in mouse ES cells

mESCs are rapidly dividing cells in which the RB family proteins are  constitutively hyperphosphorylated due to constitutive Cyclin-dependent kinase (Cdk) activity^25^. Thus, the repressor E2Fs, including E2F4, are expected to be inactive in mESCs, with either low expression levels or largely cytoplasmic localization. However, we noticed that E2F4 is the most highly expressed E2F in mESCs both at the RNA (ENCODE data^26^, Supplementary Fig. 1a) and the protein level^27^ (Supplementary Fig. 1b). Furthermore, previous chromatin immunoprecipitation (ChIP) studies revealed that ectopically-expressed E2F4 can regulate numerous loci in the genome of mESCs, including genes coding for histones^28^, often in conjunction with the cell cycle activator c-MYC^29^, suggesting that E2F4 can be nuclear and access chromatin in mESCs. An analysis of these ChIP datasets showed enrichment for biological processes normally regulated by the E2Fs (cell cycle, DNA repair, and metabolism) (Supplementary Fig. 1c, d). These observations led us to further investigate E2F4 activity in mESCs.

### E2F4 is not required for the self-renewal of mouse ES cells

We generated E2F4 knockout (E2F4KO) mESCs using CRISPR/Cas9 with independent single-guide RNAs (sgRNAs) to target exons 1 and 3 of the *E2f4* locus in J1 mESCs (Supplementary Fig. 2a). E2F4 knockout in independently-derived clonal lines was confirmed at the protein level and by sequencing of the allelic regions around the sgRNA targets (Supplementary Fig. 2b,c). Control clones included mESC clones that had been transfected with the same Cas9 and sgRNA system but retained E2F4 protein (WT, wild-type), as well as mESC clones transfected with Cas9 only (see Methods).

Repressor complexes comprised of E2F4 and RB family proteins have been shown to control the expression of pluripotency programs^30^. We therefore first sought to determine whether loss of E2F4 affects the pluripotency of mESCs. E2F4KO and WT mESC lines were morphologically indistinguishable and could be kept in culture over 20 passages. Low-density plating followed by staining for alkaline phosphatase (AP), a marker of undifferentiated mESCs, revealed no significant difference between the clonogenic ability of E2F4KO and WT mESCs, even at high passage numbers (Supplementary Fig. 3a-c). Both E2F4KO and WT mESCs could be differentiated into embryoid bodies that gave rise to neurons (Supplementary Fig. 3d). In addition, both E2F4KO and WT mESCs gave rise to teratomas in nude mice with evidence of multi-lineage differentiation (Supplementary Fig. 3e). Thus, E2F4 does not critically maintain the self-renewal or pluripotency of mESCs and does not play a critical role in the ability of these cells to differentiate in the contexts analyzed.

### E2F4 is required for the optimal growth of mouse ES cell populations

**Fig. 1:**
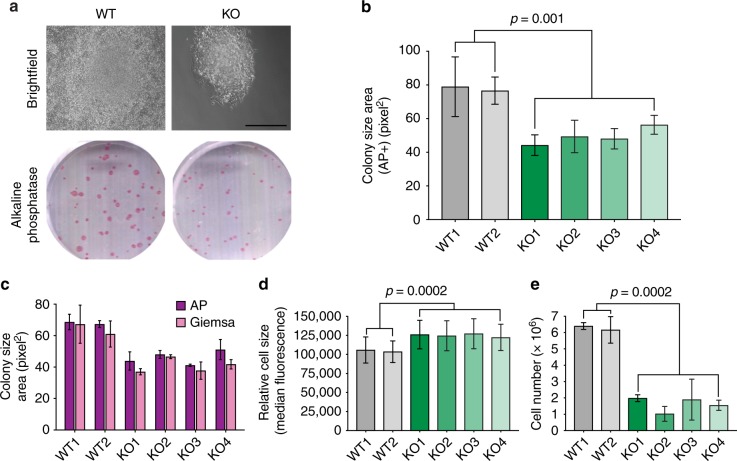
Loss of E2F4 leads to defects in mouse ES cell growth. **a** Representative brightfield images (scale bar, 400 µm) and alkaline phosphatase (AP) staining (wells from a 6-well plate are shown) of wild-type (WT) and E2F4KO (KO) colonies one week after plating single cells (*n* > 10 assays per genotype). **b** Quantification of the size of AP^+^ colonies (unpaired *t*-test; *n* = 3 biological replicates per clone). **c** Comparison of the area of AP staining (undifferentiated cells) versus Giemsa staining (all cells) in an independent set of colonies (*n* = 3 biological replicates per clone) (no significant differences). **d** Size of WT and E2F4KO individual cells as estimated by forward scatter in flow cytometric analysis (unpaired *t*-test; *n* = 2 biological replicates per clone). **e** Total number of cells per 10 cm dish one week after plating at low density (unpaired *t*-test; *n* = 3 biological replicates per clone). Data shown as the mean and standard error of the mean

In prolonged culture, we noticed that E2F4KO mESCs grow more slowly compared to their WT counterparts (Supplementary Fig. 3a). In addition, E2F4KO mESCs formed significantly smaller AP-positive (AP^+^) colonies when plated at low density (Fig. 1a, b). We observed the same phenotype with E2F4KO mESCs from a different line (R1 mESCs) that we generated using the same CRISPR/Cas9 approach (Supplementary Fig. 4a, b). The difference could also be observed when mESCs were plated in 2i media (Supplementary Fig. 4c), which supports a more naïve/ground pluripotent state^31^. The formation of smaller AP+ colonies was not due to an increased tendency of E2F4KO mESCs to differentiate at low density, as the area stained by Giemsa (which labels all cells) was equivalent to the area of AP staining (Fig. 1c). Moreover, the decrease in colony size was not due to a decrease in cell size, as E2F4KO mESCs were larger than WT mESCs as determined by flow cytometry (Fig. 1d), but to a decrease in the number of cells within the colonies (Fig. 1e).

The loss of all three RB family proteins (RB, p107, and p130), which normally repress the G1/S transition in a complex with E2F4, has little or no reported effect on the expansion of mESCs^32,33^. These data showing that E2F4 is required for the normal expansion of mESCs suggested that E2F4 may have new roles in mESCs beyond acting as a repressor of the G1/S transition.

### E2F4 drives the cell cycle progression of mouse ES cells

**Fig. 2:**
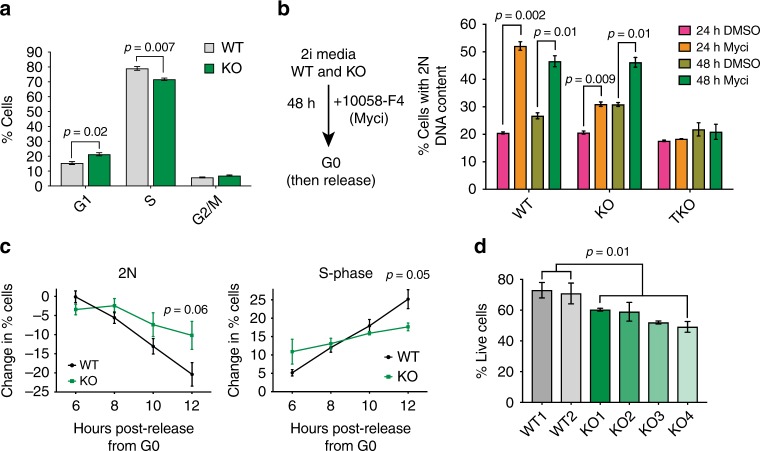
E2F4 mutant mouse ES cells are defective for cell cycle and cell survival. **a** Quantification of the percentage of cells in G1, S, and G2/M phases based on BrdU/PI FACS analysis (unpaired *t*-test; *n* = 2 biological replicates with 2 wild-type (WT) and 4 E2F4KO (KO) clones), performed after 4 days of low density plating. **b** Schematic of synchronizing mESCs in G0 using the Myc inhibitor (Myci) 10085-F4 (left), and quantification of the percentage of cells in G0/G1 in WT, E2F4KO (KO), and RB family triple knockout (TKO)  populations at 24 and 48 h of treatment (unpaired *t*-test; *n* = 2–3 biological replicates with 2 clones of each genotype). **c** Quantification of the percentage of arrested and cycling cells post-release from Myci. Cell cycle structure was measured with BrdU/PI staining 6, 8, 10, and 12 h after withdrawal of Myci from the media (unpaired t-test was performed with all individual data points from *n* = 3–4 biological replicates with 1–2 clones of each genotype). **d** Quantification of the percentage of AnnexinVneg/PIneg cells (live cells) in WT and E2F4KO populations 4 days after low density plating (unpaired *t*-test; *n* = 3 biological replicates per clone). Data shown as the mean and standard error of the mean

We next analyzed the cell cycle structure and cell survival of WT and E2F4KO mESCs. E2F4KO populations had a greater percentage of cells in G1 and a smaller percentage of cells in S phase (Fig. 2a and Supplementary Fig. 5a). To probe for possible defects at specific cell cycle stages in the E2F4KO cells, we synchronized mESCs in G0 using the Myc inhibitor 10058-F4, which can arrest mESCs in G0 without compromising their self-renewal and pluripotency^34^, and measured the kinetics of cell cycle entry upon release from inhibition. Both WT and E2F4KO mESCs were similarly able to exit the cell cycle and maintain viability after 48 h of Myc inhibition, as recently described for WT mESCs, while RB family TKO mESCs are unable to arrest upon Myc inhibition (Fig. 2b and Supplementary Fig. 5b). Upon inhibitor withdrawal and cell cycle re-entry, the percentage of G0/G1 cells decreased more slowly in the E2F4KO mESC populations, while the percentage of S phase cells increased more slowly as well (Fig. 2c). These results suggest that E2F4 is a positive regulator of progression through the G1/S phase of the cell cycle in mESCs. In addition to cell cycle defects, we observed decreased viability in low-density assays for E2F4KO mESCs compared to their controls (Fig. 2d and Supplementary Fig. 5c), which may contribute to the diminished expansion of E2F4KO mESCs in culture.

### E2F4 mutant mouse ES cells have lower expression of cell cycle genes

**Fig. 3:**
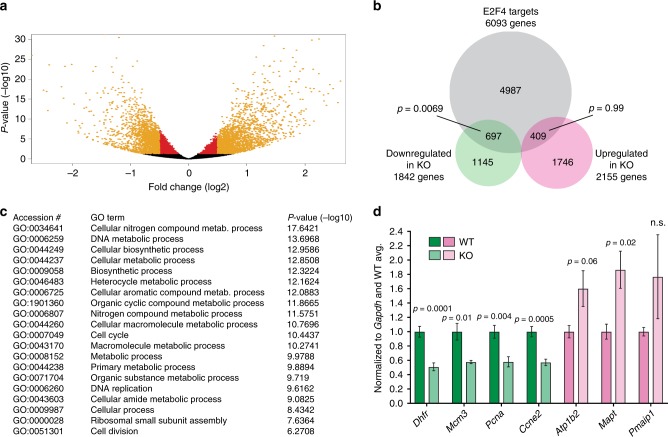
Loss of E2F4 leads to genome-wide changes in gene expression. **a** Volcano plot of changes in gene expression upon E2F4 loss in mouse ES cells; y-axis represents -log10 transformation of *p*-value and *x*-axis represents log2 transformation of fold change. Red circles represent significantly changed genes (*q*-value < 0.05) while orange circles represent significantly changed genes with a fold change > 0.5 (log2). **b** Overlap between E2F4 targets in mouse ES cells  and genes differentially expressed in E2F4KO (KO)cells with respect to wild-type. **c** GO terms for biological processes enriched in genes downregulated in E2F4KO cells (*q*-value < 0.05, fold change > 0.5 (log2)). GO terms were filtered for redundancy through REVIGO and the top 20 most significant are shown. **d** RT-qPCR validation of differentially expressed genes. Expression of downregulated genes in WT (dark green) and E2F4KO cells (light green); and upregulated genes in WT (pink) and E2F4KO cells (light pink), was normalized to *Gapdh* expression and then to expression levels in WT cells (unpaired *t*-test was performed with all individual data points from *n* = 2–4 biological replicates with 2 WT and 2 E2F4KO clones). Data shown as the mean and standard error of the mean

To identify the molecular changes that underlie the defects we observed in E2F4KO mESCs, we performed an RNA-Seq analysis of WT and E2F4KO mESCs. This analysis revealed that 1842 and 2155 genes were significantly downregulated and upregulated, respectively, upon loss of E2F4 (adjusted *p*-value < 0.05, absolute log2(fold-change) >0.5) (Fig. 3a, Supplementary Fig. 6a and Supplementary Data 1). By Gene Set Enrichment Analysis (GSEA), the downregulated genes enriched for a larger number of hallmark gene sets than the upregulated genes. Such hallmark gene sets include MYC and E2F targets and other genes involved in cell cycle pathways (Supplementary Fig. 6b). The proximal regulatory regions of the downregulated genes were also enriched for E2F binding sites (Supplementary Fig. 6c and Supplementary Data 2-3).

We next integrated our RNA-Seq datasets with existing ChIP datasets of ectopically-expressed E2F4 in mESCs^29^. 697 out of 1842 downregulated genes and 409 out of 2155 upregulated genes overlapped with E2F4 direct targets from the ChIP data (Fig. 3b and Supplementary Data 4). While the overlap between the downregulated genes and the ChIP datasets was significant (*p* = 0.0069), the overlap between the upregulated genes and the ChIP datasets was the same as would be expected from two random gene sets of the same size (*p* = 0.99), suggestive of an enrichment for direct E2F4 targets in the downregulated group. Downregulated genes bound by E2F4 were enriched for biological processes that involve metabolic, biosynthetic, and cell cycle genes (Fig. 3c). Notably, RT-qPCR analysis of canonical E2F targets (*Dhfr*, *Mcm3*, *Pcna*, *Ccne2*) in an independent passage of E2F4KO and WT mESCs confirmed that the expression of these genes is downregulated upon loss of E2F4 expression (Fig. 3d). Upregulated genes are enriched for metabolic processes as well, and also for genes involved in development and cellular adhesion (Supplementary Fig. 6d).

These gene expression and ChIP data indicate that E2F4 serves as both a direct activator and a direct repressor of gene programs in mESCs, with a more prominent role as an activator. This includes a direct role as an activator of genes implicated in cell cycle progression that are usually upregulated by the canonical activator E2Fs in other cell types.

### E2F4 activator function relies on its transactivation domain

**Fig. 4:**
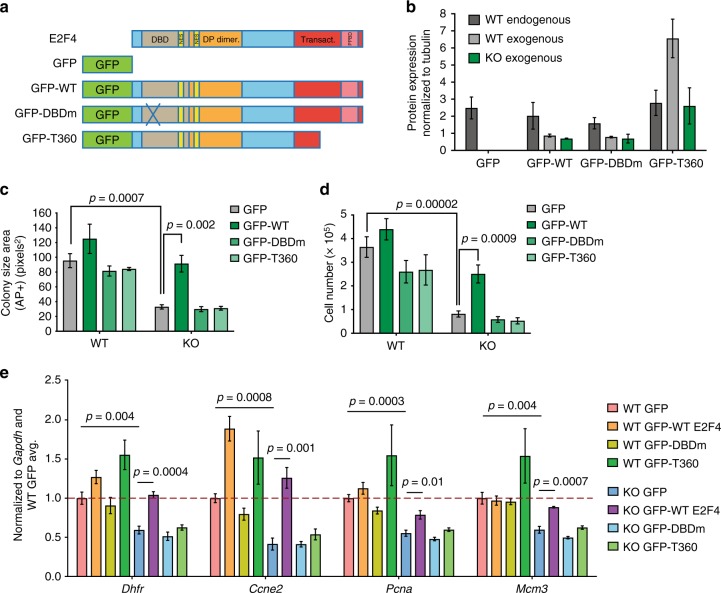
The transactivation and DNA binding domains of E2F4 are required for its function in mouse ES cells. **a** Schematic of the design of E2F4 mutant constructs. All constructs were fused C-terminal to a GFP tag. Wild-type (WT) E2F4 contains a DNA binding domain (DBD), a dual nuclear export signal (NES), a DP dimerization domain, a transactivation domain, and an RB family/pocket proteins binding domain (PPBD). GFP-DBD contains three point mutations that make contacts with the E2F consensus binding motif and the DP dimerization partners. GFP-T360 is a truncation mutant that lacks the last 50 amino acid residues, inactivating the transactivation domain. **b** Quantification of endogenous and exogenous E2F4 expression by immunoassay (from fluorescence units) in WT (gray) and E2F4KO (KO, green) cells (*n* = 2 biological replicates with 1 WT and 1 E2F4KO clone). **c** Quantification of colony size by AP staining (unpaired t-test was performed with all individual data points from *n* = 2 biological replicates with 2 WT and 2 E2F4KO clones in each replicate) and **d** Total number of cells per well in 6-well plates one week after plating at low density (unpaired t-test was performed with all individual data points from *n* = 4 biological replicates with 2 WT and 2 E2F4KO clones in each replicate). **e** RT-qPCR analysis of E2F4 targets and canonical cell cycle genes. Expression of genes in WT and E2F4KO cells expressing each of the constructs, was normalized to *Gapdh* expression and then to expression levels in WT cells expressing GFP (unpaired *t*-test was performed with all individual data points from *n* = 2 biological replicates with 2 WT and 2 E2F4KO clones in each replicate). Data shown as the mean and standard error of the mean

All of the E2Fs that physically interact with the RB family proteins, including E2F4, have a C-terminal transactivation domain (Fig. 4a). However, E2F4 also contains a dual nuclear export signal, and its translocation into the nucleus relies on RB family binding, which inhibits the transactivation domain^9,11,12,35,36^. Based on our data, we sought to further test the possibility that E2F4 may act as a direct transcriptional activator in mESCs. To this end, we expressed wild-type and mutant forms of E2F4 (all fused to GFP) in WT and E2F4KO mESCs at levels similar or lower to endogenous E2F4, and analyzed the ability of these constructs to rescue colony size and gene expression (Fig. 4a, b and Supplementary Fig. 7a). GFP-T360 harbors a truncation of the transactivation domain and is incapable of inducing target gene expression^36^. GFP-DBD harbors point mutations in the DNA binding domain that disrupt interactions with the E2F4 consensus binding sequence and the DP family proteins^37^. The exogenous wild-type E2F4 and truncated GFP-T360 proteins localized to the nucleus, but the GFP-DBD mutant was less present in the nucleus (Supplementary Fig. 7b). Importantly, ectopic expression of E2F4 in E2F4KO mESCs restored the size of AP^+^ colonies to wild-type levels and increased cell count after one week, while expression of the mutants had no effect (Fig. 4c, d). Expression of cell cycle genes was similarly rescued to wild-type levels by wild-type E2F4 (Fig. 4e), but not by the E2F4 mutants. Notably, expression of the E2F4 constructs in WT mESCs did not significantly impact cell proliferation and the expression of cell cycle genes, suggesting that these constructs do not have nonspecific effects that may affect the results of the rescue experiments. GFP-T360 is expressed at slightly higher levels than the other constructs in WT mESCs and is associated with a trend toward increased expression of cell cycle genes, likely due to the displacement of endogenous repressor complexes from the promoters of these genes. However, the change in gene expression was not significant for the majority of genes tested. The inability of the overexpressed E2F4 mutants to rescue these phenotypes supports the idea that E2F4 serves as a direct activator of target genes, including cell cycle genes, in mESCs.

E2F transcription factors often compensate for each other. We found no evidence of increased transcription for E2F1/2/3, but observed a significant increase in the binding of E2F1 to canonical cell cycle genes in E2F4KO mESCs (Supplementary Fig. 8a, b), supporting the idea of at least partial compensation. However, we were unable to test whether loss of E2F1 would enhance the phenotypes observed in E2F4KO cells due to the rapid loss of E2F1 mutant mESC clones (Supplementary Fig. 8c).

### The function of E2F4 in mouse ES cells is RB family-independent

**Fig. 5:**
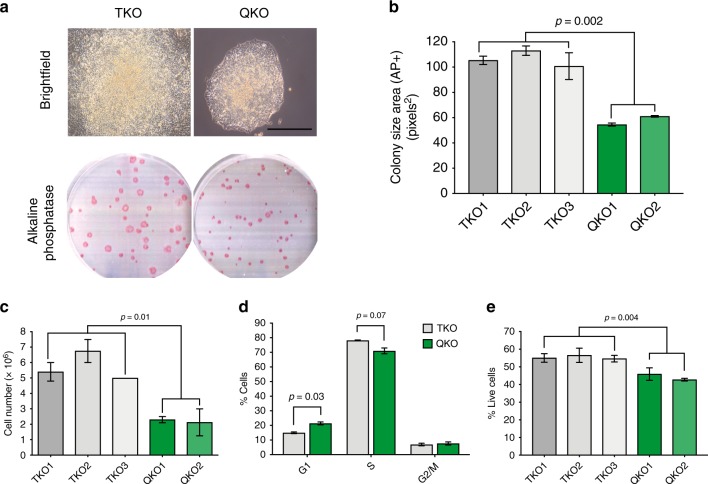
Loss of E2F4 leads to defects in the growth of RB family TKO mouse ES cells. **a** Representative brightfield images (scale bar, 400 µm) and alkaline phosphatase (AP) staining (wells from a 6-well plate are shown) of RB family triple knockout (TKO) and RB family knockout, E2F4KO (QKO) colonies one week after plating single cells (n > 10 assays per genotype). **b** Quantification of the size of AP^+^ colonies (unpaired *t*-test; *n* = 2 biological replicates per clone). **c** Total number of cells per 10 cm dish one week after plating at low density (unpaired *t*-test; *n* = 2 biological replicates per clone). **d** Quantification of the percentage of cells in G1, S, and G2/M phases based on BrdU/PI FACS analysis (unpaired *t*-test; *n* = 2–3 biological replicates with 2 TKO and 2 QKO clones), performed 4 days after low density plating. **e** Quantification of  the percentage of AnnexinVneg/PIneg cells (live cells) in TKO and QKO populations 4 days after low density plating (unpaired *t*-test; *n* = 2 biological replicates per clone). Error bars represent ± s.e.m. Data shown as the mean and standard error of the mean

RB family proteins are hyperphosphorylated and inactive in mESCs^32^, suggesting that the function of E2F4 in this context may be in part RB family-independent. To test this idea, we knocked out E2F4 in RB family TKO mESCs to generate *E2f4*^*−/−*^*;Rb*^*−/−*^*;p130*^*−/−*^*;p107*^*−/−*^ (quadruple knockout, QKO) mESCs (Supplementary Fig. 2d, e). Similar to its effects in WT mESCs, loss of E2F4 led to smaller AP^+^ colonies with fewer cells in QKO MESCs compared to TKO controls (Fig. 5a–c). Accordingly, loss of E2F4 in TKO mESCs led to a greater percentage of QKO mESCs in G1 and a smaller percentage in S phase (Fig. 5d), as well as decreased viability (Fig. 5e).

**Fig. 6:**
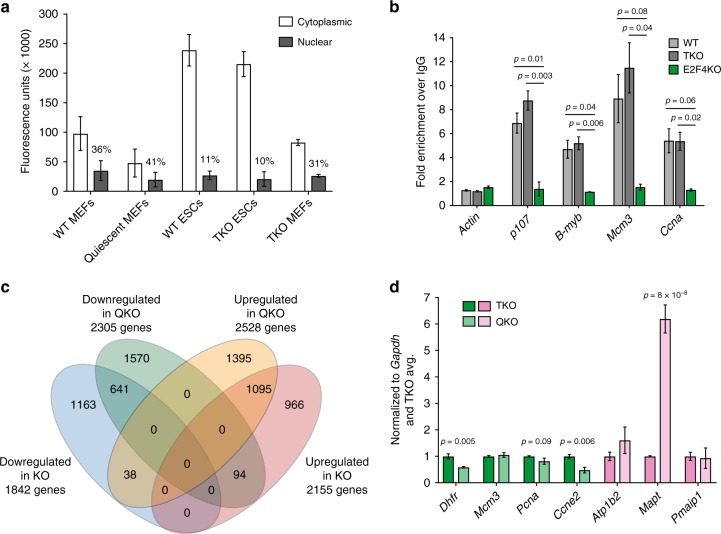
E2F4 can access chromatin and regulate target genes in an RB family-independent manner. **a** Quantification of cytoplasmic and nuclear E2F4 expression by immunoassay in cycling and quiescent mouse embryonic fibroblasts (MEFs), WT and TKO mouse ES cells, and TKO MEFs (*n* = 2–3 biological replicates per cell type). The percentage of nuclear E2F4 is shown. See Supplementary Fig. 9 for fractionation controls. **b** Quantification of E2F4 binding to target genes and an *Actin* negative control in WT, TKO, and E2F4KO mouse ES cells, assayed by ChIP-qPCR (unpaired *t*-test was performed with all individual data points from *n* = 2–3 biological replicates of 2 WT, 2 TKO, and 1 E2F4KO clone(s)). Binding was normalized to 10% input and then to binding of an IgG control. **c** Overlap of genes that are differentially expressed in QKO versus TKO mouse ES cells, and genes that are differentially expressed in E2F4KO (KO) versus wild-type (WT) mouse ES cells (*q*-value < 0.05 with a fold change > 0.5 (log2)). The sets of upregulated (1095) and downregulated (641) genes between E2F4KO/WT and QKO/TKO mouse ES cells are highly similar (*p*-value close to zero). **d** RT-qPCR validation of differentially expressed genes. Expression of downregulated genes in TKO (dark green) and QKO mouse ES cells (light green); and upregulated genes in TKO (pink) and QKO mouse ES cells (light pink), was normalized to *Gapdh* expression and then to expression levels in TKO cells (unpaired *t*-test was performed with all individual data points from *n* = 2–4 biological replicates with 2 TKO and 2 QKO clones). Data shown as the mean and standard error of the mean

The complete absence of the RB family proteins had no effect on the ability of E2F4 to translocate into the nucleus in mESCs (Fig. 6a, and Supplementary Fig. 9 for loading controls). In comparison to mouse embryonic fibroblasts (MEFs), WT and TKO mESCs have a relatively lower percentage of nuclear E2F4; however, the absolute amount of nuclear E2F4 in mESCs is similar to that of quiescent MEFs, where E2F4 is expected to be predominantly bound to chromatin to silence cell cycle genes. In addition, loss of RB family proteins did not affect the binding of E2F4 to the promoter region of canonical cell cycle genes in mESCs (Fig. 6b). Furthermore, RNA-Seq and RT-qPCR analyses of TKO and QKO mESCs showed a significant overlap between downregulated and upregulated genes and their cellular functions with data from the WT and E2F4KO comparison (Fig. 6c, d, Supplementary Fig. 6e, and Supplementary Data 5).

These genetic experiments demonstrate that a large part of the cellular and molecular phenotypes due to loss of E2F4 in mESCs is independent of the RB family. These observations are consistent with the hyperphosphorylation of RB family proteins in mESCs and a lack of physical interaction between these proteins and E2F4. Notably, some of the repressor activity of E2F4 in mESCs does not require the RB family. Similarly, direct binding to and activation of some canonical cell cycle genes by E2F4 is also independent of the RB family.

### E2F4 activates transcription with chromatin modifying factors

**Fig. 7:**
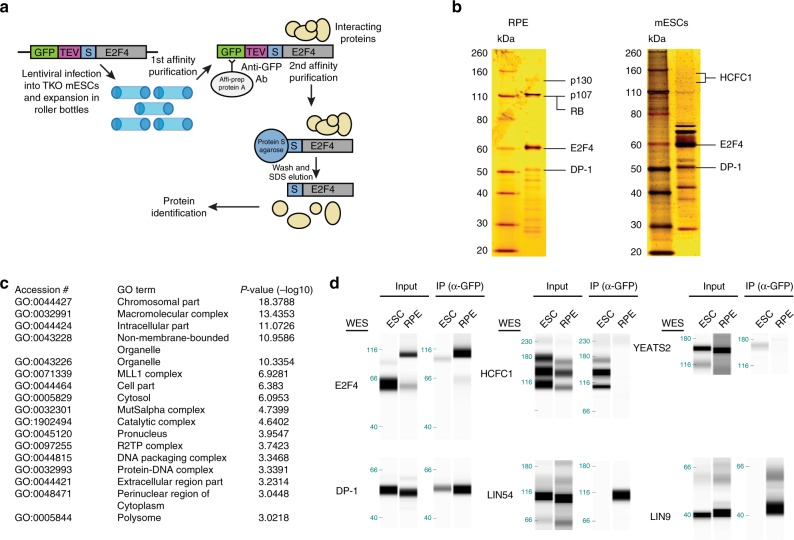
Transcriptional activation by E2F4 in mouse ES cells is mediated by chromatin modifiers. **a** Schematic representation of the affinity purification-mass spectrometry approach to identify the E2F4 interactome. **b** Silver stain of eluted fractions from RPE cells and mouse ES cells . Slices of gel were subjected to mass spectrometry, and the indicated protein bands were identified by analyzing the proteins enriched in each slice. **c** GO terms for cellular components enriched in the list of mouse ES cell-specific candidate interactors. **d** Validation of interactions between GFP-E2F4 and DP-1, HCFC1, YEATS2, LIN54, and LIN9, by co-immunoprecipitation followed by immunoassay, in mouse ES cells and human RPE cells. Transcriptional activators (HCFC1 and YEATS2) preferentially bind to E2F4 in mouse ES cells while members of the DREAM repressor complex (LIN54 and LIN9) bind preferentially to E2F4 in RPE cells. Molecular weights (kDa) are indicated on the left side (one experiment shown of at least two experiments)

The identification of RB family-independent roles for E2F4 in mESCs suggested that E2F4 might participate in novel, RB family-independent protein complexes that can contribute to gene regulation. To test this hypothesis and to identify novel cofactors that might allow E2F4 to control transcriptional programs in the absence of functional RB family proteins, we expressed a GFP- and S-tagged (localization and affinity purification tag, LAP-tag)^38^ lentiviral E2F4 construct in TKO mESCs, pulled down the E2F4 protein, and performed mass spectrometry on the eluted fraction (Fig. 7a, b). This approach revealed 108 candidate interactors (Supplementary Data 6). We performed the same experiment in RPE cells, human somatic cells with a slower cell cycle structure and active RB. E2F4 bound to known partners, including RB and members of the DREAM repressor complex^39^ in RPE cells (Supplementary Data 6) and bound to the DP family proteins similarly in both cell types (Supplementary Data 7). Strikingly, 95 of the 108 candidate E2F4 interactors in TKO mESCs were specific to mESCs, and a number of these factors are not reported to associate with any of the E2Fs (Supplementary Fig. 10a, b and Supplementary Data 6). Some post-translational modifications on E2F4 were different between the two cell types but the biological significance of these modifications remains unclear (Supplementary Data 8).

A GO term analysis for the mESC-specific interactors identified an enrichment for nuclear and DNA binding complexes, including components of the MLL1 and SAGA complexes involved in transcriptional regulation and the MutS-alpha complex involved in DNA repair (Fig. 7c). Members of chromatin modifying complexes, such as HCF-1, WDR5, and TRRAP, are known to contribute to the transcriptional activation of E2F targets or cell cycle genes in certain contexts^36,40,41^. We validated through co-IP that E2F4 binds to HCF-1 and YEATS2 (a member of the ATAC histone acetyltransferase complex^42^) robustly and specifically in TKO mESCs, while binding of E2F4 to DREAM complex members (LIN54 and LIN9) was specific to RPE cells, and binding of E2F4 to DP-1 was similarly robust between the two cell types (Fig. 7d).

Based on these observations, we hypothesized that mESC-specific interactors may serve as co-activators of E2F4 to activate the transcription of E2F targets in mESCs. We performed rescue experiments in E2F4KO mESCs using a previously described mutant form of E2F4 with point mutations that abrogate binding to HCF-1^40^. Overexpression of this HCF-1 binding domain mutant form of E2F4 resulted in a phenotype similar to cells expressing wild-type E2F4, when compared to cells expressing the T360 truncation of E2F4 (which behaves similar to a GFP-expressing control—Fig. 4d) (Supplementary Fig. 11a). Overexpression of a form of E2F4 harboring mutations within the RB family binding domain (see Methods) was similarly able to rescue cell proliferation when compared to the truncation mutant, as expected based on our observations in TKO mESCs (Supplementary Fig. 11a)^40,43^. These observations suggest that the interaction between E2F4 and HCF-1 is not absolutely required for E2F4 to function as a transcriptional activator in mESCs, and that multiple activator complexes may contribute to this role.

The p300 and CBP (CREB-binding protein) proteins were not identified in the mass spectrometry analysis. However, they constitute an important family of acetyltransferases involved in the regulation of gene expression and cell proliferation, including the regulation of targets of activator E2Fs^44–46^. We examined binding of purified recombinant E2F4 transactivation domain to three commonly accessed protein interaction domains in CBP (KIX, TAZ1, and TAZ2). Using isothermal titration calorimetry, we observed the most robust binding signal using TAZ1 (which constitutes an interaction site for a number of transcription factors^47^) (Supplementary Fig. 11b and data not shown). These data further support the idea that E2F4 can directly recruit transcriptional activators, similar to activator E2Fs.

**Fig. 8:**
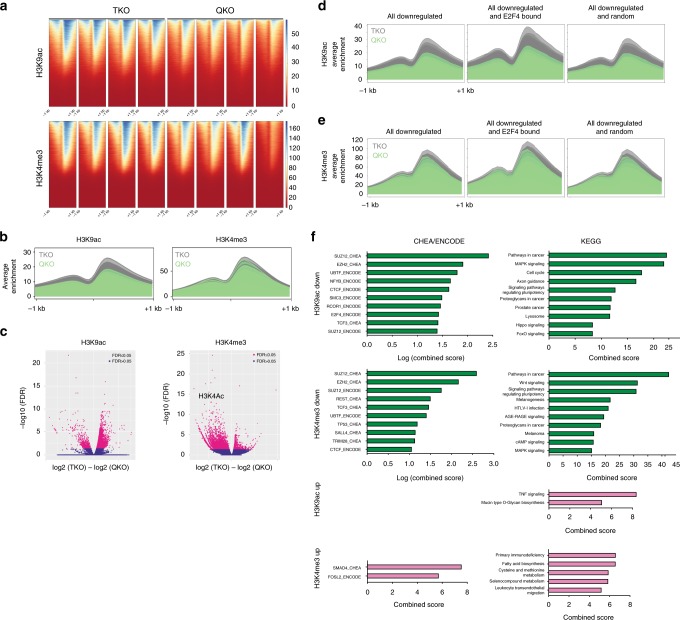
ChIP-seq analysis of the role of E2F4 in gene activation in mouse ES cells. **a** Heatmap of enrichment scores of H3K9ac and H3K4me3 ChIP-seq signal across transcription start sites (TSS, −1/ + 1 kb) in 2 biological replicates of 2 TKO and 2 QKO clones (biological replicates are next to each other). **b** Average enrichment of H3K9ac and H3K4me3 signal around TSS (-1/ + 1 kb) in TKO and QKO clones. **c** Volcano plot showing genome-wide comparison of detected peaks in TKO and QKO cells for H3K9ac and H3K4me3. **d**, **e** Average enrichment of H3K9ac (**d**) and H3K4me3 (**e**) signal around TSS (−1/+1 kb) comparing all downregulated genes in QKO cells compared to TKO cells, genes that are downregulated and bound by E2F4, and a random set of downregulated genes. **f** Enrichr analysis of CHEA/ENCODE motifs and KEGG pathways significantly (*p* < 0.05) enriched in genes with downregulated and upregulated H3K9ac and H3K4me3 signal upon E2F4 loss. Top motifs/pathways (by combined score) are shown

To further investigate the mechanisms by which E2F4 may act as a direct activator of transcription independently of the RB family, we performed ChIP-seq assays in TKO and QKO mESCs for H3K4me3 and H3K9ac, two chromatin marks associated with transcriptional activation. We chose these marks on the basis of their connection to the E2F4 interactome: H3K4 trimethylation is mediated by the MLL complex, which contains the E2F4 interactors HCF-1 and WDR5^40^; H3K9 acetylation is mediated by the SAGA complex, which contains TRRAP, and by the ATAC complex, which contains YEATS2, GCN5, and WDR5^42^. Indeed, GCN5 and ectopically-expressed E2F4 both associate with MYC in mESCs^48^ and we found a significant overlap between GCN5 and E2F4 targets in these cells (Supplementary Fig. 12a). H3K4me3 and H3K9ac signals showed the expected distribution around transcriptional start sites (TSS) (Fig. 8a). We observed a clear decrease of H3K9ac around TSS in QKO compared to TKO mESCs, while the difference in H3K4me3 signal between QKO and TKO cells was less apparent (Fig. 8a, b). When compared to the corresponding peaks in TKO mESCs, a majority of H3K9ac peaks in QKO mESCs showed a decrease in signal strength (10008 vs. 676 peaks with increased signal), whereas a majority of H3K4me3 peaks gained signal (5294 vs. 1679 peaks with decreased signal) (Fig. 8c, Supplementary Fig. 12b, and Supplementary Data 9-12). Notably, only 6% and 4% of peaks that gained signal for H3K4me3 or H3K9ac, respectively, in QKO mESCs relative to TKO mESCs overlap with TSS (here defined as −5 kb/+1 kb). In contrast, almost 50% of peaks that lose signal for H3K4me3 or H3K9ac overlap with TSS (47% and 48%, respectively).

Collectively, these data suggest that loss of E2F4 is associated with a global decrease in H3K9ac and H3K4me3 around gene promoters, consistent with the proposed role of E2F4 as a transcriptional activator at proximal regulatory regions in mESCs. Additionally, these data suggest that loss of E2F4 has a greater impact on H3K9ac rather than H3K4me3 levels, and thus that histone acetylation may be a more significant contributor to E2F4 activity in mESCs.

When we integrated our ChIP-Seq and RNA-Seq data, we found that genes that were downregulated upon E2F4 loss show stronger loss of H3K4me3 and H3K9ac signals than those that were upregulated (Supplementary Fig. 12c,d). Of these downregulated genes, those bound by E2F4 were enriched for genes with higher H3K9ac levels and slightly higher H3K4me3 levels, when compared to all downregulated genes or a random set of downregulated genes (Fig. 8d, e and Supplementary Fig. 12e, f). Notably, an analysis of the genes associated with decreased H3K9ac in their promoter region identified an enrichment for genes bound by E2F4 and cell cycle genes. As few genes were associated with either increased H3K4me3 or H3K9ac, an enrichment analysis of these genes yielded very few significantly (*p* < 0.05) enriched motifs or pathways (Fig. 8f). These data suggest that while loss of E2F4 results in a global decrease of acetylation, this decrease is more significant at direct E2F4 targets, including cell cycle genes. Taken together with in vitro binding data and the proteomics analysis, these data support a model in which E2F4 recruits components of histone acetyltransferase complexes to mediate transcriptional activation of cell cycle genes and other E2F targets in mESCs.

## Discussion

E2F transcription factors not only regulate the cell cycle in conjunction with RB family proteins, but also play roles in apoptosis, differentiation, and stem cell biology that are E2F- and cell type-specific^4,49^. Identifying these roles is critical for a better understanding of how cell fate decisions are made in development and misregulated in disease. Here we provide evidence that E2F4 regulates the transcription of a large number of genes in mESCs, including the activation of cell cycle genes, in a manner that is largely independent of the RB family. Our studies provide new insight into the activity of a key E2F family member in a cell type relevant to development and cancer.

Our data show that E2F4 has functions that are independent of the RB family. These observations raise several questions, including how E2F4 is recruited to the nucleus and the DNA. Ectopic expression of DP-2 and DP-3, which heterodimerize with E2Fs, can be sufficient to translocate E2F4 to the nucleus^17,35,50^. DP-2, which associates with E2F4 in mESCs^29^ and bears a nuclear localization signal (NLS)^51^, may be implicated in E2F4 nuclear translocation in mESCs. Our data also indicate that E2F4 interacts with proteins involved in nuclear import (KPNB1, RanGAP1, and RanBP2), which may help promote E2F4 nuclear translocation in mESCs. Finally, E2F4 harbors a weak putative NLS in amino acids 52-61^51^ and E2F4 may translocate into the nucleus in a cofactor-independent manner, similar to E2F5 during keratinocyte differentiation^52^. Consistently, our DNA binding domain mutant, which harbors point mutations within this putative NLS (R56A and R57A), was unable to translocate into the nucleus. It will therefore be interesting to examine the subcellular localization of the N-terminus of E2F4, as well as of different mutants containing point mutations within and outside the putative NLS.

E2F4 has a transactivation domain and it is not surprising that E2F4 activates the transcription of genes once it is bound to regulatory regions, as shown in overexpression experiments^53^. One question remains as to whether E2F4 interacts with the same transcriptional cofactors as the classical activator E2Fs when it acts as a transcriptional activator. Our interactome data in TKO mESCs identified several candidate interactors of E2F4 that mediate the transcriptional activity of E2F1 to drive diverse biological processes. These interactors include HCF-1, a component of the MLL complex^40^, as well as TRRAP^36,41^ and Pontin/Reptin^54^. Our analysis of the mass spectrometry data does not show any post-translational modifications that may mediate specific binding to these factors. Possibly, these factors bind to E2F4 in mESCs because the domain of E2F4 normally bound by the RB family proteins is available for interaction with other partners. In our interactome analysis, we did not identify Multicilin or GEMC1, which have been found to function with E2F4 to activate transcription^18,19^, likely because the genes coding for these proteins are expressed at a low level in mESCs. Our ChIP-seq studies strongly suggest that histone acetyltransferase complexes are implicated in E2F4 activity in mESCs. The ATAC and SAGA complexes deposit H3K9ac and together contain several candidate E2F4 interactors, including TRRAP and GCN5, which are required for E2F transcriptional activity in other contexts^55^. Our in vitro binding data demonstrate that the E2F4 transactivation domain can directly bind to the TAZ1 domain of CBP, suggesting that CBP and/or p300 may also play a role in E2F4-dependent gene activation in some contexts. Thus, factors involved in histone acetylation may be critical partners of E2F4 in the activation of gene programs.

Our gene expression data and rescue experiments also support the idea that E2F4 may act as a transcriptional repressor even in the absence of RB, p107, and p130. Repressors such as TRIM28^28,56^ may contribute to this repression activity. Because E2F4 is an ancestral E2F and is expressed in organisms that have only one E2F homolog^57^, this switching between gene activation and gene repression may have been required for the RB/E2F pathway to serve many different functions throughout development. While E2F5 is also known as a canonical repressor E2F, it may act as a transcriptional activator in specific contexts^20,58^. However, our initial experiments did not identify a key role for E2F5 in the growth of mESCs, possibly because of lower levels of expression.

Because the transactivation domain of E2F4 is normally inhibited upon RB family binding, we speculate that the pro-proliferative role of E2F4 may be restricted to cell types where the RB family proteins are inactive, as in rapidly cycling progenitor cells and cancer cells. Several cancer types exhibit *E2F4* amplification or overexpression^5,14^. Expansion of CAG repeats in the *E2F4* coding sequence is also frequent in tumors with genomic instability such as colorectal carcinomas, and could be oncogenic by stabilizing E2F4^59^. E2F4 has also been found with the MYC transcriptional activator at regulatory regions of genes involved in cancer development^60^. In many of these experiments, however, it has been difficult to determine if the effects observed are a direct result of gene regulation by E2F4. Our work demonstrates that E2F4 directly promotes the transcription of key cell cycle genes, and we show that loss of E2F4 directly affects the proliferation and survival of mESCs. We note, however, that these pro-proliferative or pro-tumorigenic effects of E2F4 do not have to be limited to cell cycle activation. For instance, we observed strong cytoplasmic expression of E2F4 in mESCs, and it is also possible that E2F4 has important cytoplasmic functions^61^.

Our work and other studies support a model in which E2F4 should not simply be viewed as a repressor of transcription and cell cycle progression. Future work will be needed to identify the different protein complexes that E2F4 contributes to and the plethora of cellular functions that are controlled by this major E2F family member, including in stem cells and cancer cells.

## Methods

### Cell culture

RPE-FRT9 cells (originally obtained from ATCC) were cultured in DMEM/F-12 (Thermo Fisher 12400024) supplemented with 10% FBS (Gemini 100–106), 1XGlutaMax (Thermo Fisher 35050-079) and 100 U/mL penicillin-streptomycin (Thermo Fisher 15140163).

mESC lines J1 and R1 (originally obtained from the laboratory of Rudolf Jaenisch, MIT) were maintained feeder-free on 0.2% gelatin-coated (Sigma G9382) plates in Dulbecco’s modified Eagle’s medium (Gibco SH30243.01) containing 15% fetal bovine serum (Hyclone SH30071.03; VWR Seradigm 97068-085), MEM non-essential amino acids (Gibco 11140-050), penicillin-streptomycin-glutamine (Gibco 10378-016), and 0.1 mM beta-mercaptoethanol (Gibco 21985-023), supplemented with LIF. TKO mESCs were described in^32^; an independent TKO mESC line (derived from E14 mESCs) was a kind gift from the Hein te Riele lab^33^. MEFs (mouse embryonic fibroblasts, WT and TKO) were obtained and cultured as described previously^32^. Cells were passaged enzymatically using 0.05% Trypsin-EDTA (Gibco 15400-054). For long-term passaging assays, mESCs were passaged every other day for 20 passages, plating 3.0 × 10^5^ cells per passage. Growth rate was calculated for 16 passages.

For low-density assays, single-cell suspensions of 50 mESCs/mL were plated and cultured up to 1 week. Low-density assays in 2i were performed with media containing 15% serum supplemented with 0.33 nM PD0325901 (Selleckchem S1036) and 10 nM CHIR99021 (Selleckchem S2924).

For alkaline phosphatase (AP) staining, plates were fixed with 4% paraformaldehyde, washed with a citrate solution (1:50 citrate concentrate, Sigma 3861), and incubated for 30 min in the dark in a diazonium salt solution (0.25 mg/mL Fast Violet B Salt, Santa Cruz sc-215029) containing 1:24 naphthol AS-MX phosphate alkaline (Sigma 855). The number and size of AP^+^ colonies were quantified in FIJI. Giemsa stain (Sigma GS500) was applied at 1:40 in water according to manufacturer’s instructions.

### Cell cycle and cell death assays

For analysis of cell cycle structure, mESCs were plated at low density for 4 days and pulsed with BrdU for 3 h prior to trypsinization, before BrdU and propidium iodide (PI) staining and analysis. Quantification of cell death was performed using AnnexinV-FITC and PI according to the manufacturer’s instructions (BD Biosciences). Gating is shown in Supplementary Fig. 13. Cells were quantified with a BD FACSAria™ instrument.

For synchronization in G0, mESCs were plated at 25,000 cells/24-well in media supplemented with 2i, and treated the next day with Ndiff media supplemented with 2i, LIF, and the Myc inhibitor 10058-F4 (Sigma F3680), as in ref. ^34^. After 48 h of Myc inhibition, media was replaced with Ndiff media containing 2i, LIF, and DMSO only, and cell cycle kinetics were measured by BrdU/PI analysis.

### Gene knockout and overexpression

E2F4 knockout was achieved by expressing Cas9 and sgRNAs from the pX330-U6-Chimeric_BB-CBh-hSpCas9 vector, a gift from Feng Zhang (Addgene plasmid #42230). WT mESCs were plated at 2 × 10^5^ cells/6-well and transfected the next day with pX330 (empty or with the cloned sgRNAs) and pPGK-Puro (Addgene plasmid #11349, a gift from Rudolf Jaenisch) at a ratio of 9:1, using Lipofectamine 2000 according to manufacturer’s instructions. After 24 h, puromycin selection was applied at a concentration of 2 µg/mL and continued for 48 h before cells were plated for single cell cloning. Immunoblots for E2F4 were performed to verify loss of E2F4 protein in all E2F4KO clones generated from J1 mESCs (*n* = 6) and R1 mESCs (*n* = 2). E2F4KO clones used in the low-density, cell cycle, cell viability, and RNA-Seq experiments were additionally subjected to quantitative immunoassay for E2F4 protein and TOPO sequencing of the targeted alleles. Control clones include mESCs transfected with Cas9 and sgRNAs that had retained E2F4 protein, as well as mESCs transfected with Cas9 only (empty pX330 vector). Both types of control clones were used in the RNA-Seq and long-term passaging experiments, while the remaining experiments were performed with control clones that had retained E2F4 expression after transfection with Cas9 and sgRNAs. The 5′-3′ sequences of the oligonucleotides cloned into pX330 are: E2F4-exon1-forward CACCGAGGTCAAGCACGCCGTCCT, E2F4-exon1-reverse AAACAGGACGGCGTGCTTGACCTC, E2F4-exon3-forward CACCGCAGCAACGAGAGCAAGAAC, E2F4-exon3-reverse AAACGTTCTTGCTCTCGTTGCTGC.

E2F4 knockout in TKO mESCs was achieved similarly with the same sgRNAs by transfecting TKO mESCs (E14) with pX330 and pTracer-CMV/Bsd/lacZ as above and performing selection with blasticidin at a concentration of 10 µg/mL. Controls were TKO mESCs transfected with Cas9 and sgRNAs that had retained E2F4 protein.

A similar strategy was used to knock out E2F1 using the following oligonucleotides: E2F1-exon1-forward CACCGCGGTGCACTAGGCGCGGGT, E2F1-exon1-reverse AAACACCCGCGCCTAGTGCACCGC, E2F1-exon1-forward2 CACCGCAGCACGTCAGAATCGCGA, E2F1-exon1-reverse2 AAACTCGCGATTCTGACGTGCTGC.

For the rescue experiments, vectors expressing GFP, GFP-E2F4, GFP-DBDm, and GFP-T360 were constructed by cloning cDNAs into the PiggyBac vector, PB-EF1α-MCS-IRES-GFP with a puromycin resistance gene (a kind gift from Joanna Wysocka). Correct insertion was validated using the Zero Blunt TOPO PCR cloning kit (Thermo Fisher K280020) according to manufacturer’s instructions and Sanger sequencing. WT and E2F4KO mESCs (R1) were transfected with each rescue vector and PiggyBac transposase at a ratio of 9:1 using Lipofectamine 2000, and puromycin selection was applied at 2 µg/mL.

Vectors expressing 3xFLAG-tagged E2F4 (FLAG-WT), the two mutant versions of E2F4 harboring point mutations in the HCF-1 and RB binding domains (FLAG-HCFC1m and FLAG-RBm), and truncated E2F4 (FLAG-T360) were ordered from VectorBuilder. The design of FLAG-HCFC1m was based on a previous study^40^. The design of FLAG-RBm was based on the same study^40^ which showed that E2F4 contains one interaction domain mediating binding to HCF-1 and one mediating binding to RB; and on a study which showed that the RB family binding domain is conserved across the E2Fs and that mutating D423 and F425 in human E2F1 abolishes binding of E2F1 to RB^43^. Accordingly, the homologous residues in mouse E2F4 (D401 and F403), and one additional residue in the RB binding domain (D404) were mutated to alanines without touching the HCF-1 binding domain.

### Embryoid body assay

mESCs were resuspended in media without LIF at a dilution of 1.0 × 10^5^ cells/mL, and 20 μL drops of the suspension were plated on the lids of petri dishes filled with phosphate-buffered saline. After three days, retinoic acid (Sigma R2625) was added to the drops at a final concentration of 0,5 µM and EBs were grown for another three days. EBs were then plated onto gelatinized coverslips in media without LIF for 7 days, fixed in 4% paraformaldehyde, permeabilized in 0.4% Triton X-100, and blocked in 50 mM glycine. After incubation with a Tuj1 antibody (BioLegend 802001) overnight, samples were washed, incubated with secondary antibody (Invitrogen Alexa Fluor 594) and counterstained with DAPI. Images were acquired on a Keyence BZ-X710 fluorescence microscope.

### Teratoma assay

Mice were maintained under the care of the Stanford Veterinary Services Center. All animal studies were approved by the Administrative Panel on Laboratory Animal Care at Stanford University. All relevant ethical regulations for animal testing and research were complied with. For each WT and E2F4KO mESC line, two replicates of 1.0 × 10^6^ cells were mixed with Matrigel and subcutaneously inoculated into the flanks of NOD scid gamma mice (The Jackson Laboratory #5557). Teratomas were collected after one week and fixed in 4% formaldehyde, and H&E staining was performed on sections. Histopathological analysis was performed by a certified pathologist (H.V.).

### Protein purification and protein-protein interactions

The human E2F4 transactivation domain (residues 390–407) was expressed in *E. coli* as an N-terminal GST fusion protein with a TEV cleavage site and purified, as previously described^62^. Mouse CBP TAZ1 (residues 340–439) was expressed in *E. coli* with a His-Nus-XL affinity tag. The TAZ1 domain was purified using nickel affinity resin followed by tag cleavage and further purification with Superdex 75 size exclusion chromatography. Equilibrium dissociation constants for binding were obtained using isothermal titration calorimetry (ITC) with a MicroCal VP-ITC system. Both E2F4 and CBP proteins were run over Superdex-75 into a buffer containing 25 mM HEPES pH 7.0, 200 mM NaCl, and 5% glycerol v/v. E2F4 TAD (300 µM) was titrated into TAZ1 (30 µM) at 25 °C. Data were fit using the MicroCal Origin analysis package. The concentration of the E2F4 peptide was adjusted such that the binding stoichiometry (N) was close to 1. We chose this approach of assuming the stoichiometry because we otherwise noticed unrealistic variability in the N value, which we attribute to difficulty measuring the E2F4 concentration accurately from its low extinction coefficient.

### Protein analysis

For detection of protein in whole cell lysates by quantitative immunoblot, cell pellets were prepared as described previously^32^, and samples were run on the WES (ProteinSimple) according to the manufacturer’s protocol. For fractionation experiments, cells were resuspended at 1 × 10^8^ cells/mL of buffer A and Triton X-100 was added at 0.1% final concentration. The cells were incubated on ice for 10 min, the nuclei were collected by centrifugation, and the supernatant was clarified by high-speed centrifugation. The nuclei were washed once in buffer A and lysed for 30 min with an equivalent volume of buffer B, and the nuclear fraction was subjected to 10 rounds of sonication (30 s ON 30 s OFF) to fragment the genomic DNA. 4 µL per cytoplasmic and nuclear fraction were loaded per lane of the WES. Co-immunoprecipitation was performed using the GFP-Trap system developed by ChromoTek (gta-10) according to manufacturer’s instructions. TKO mESCs were transfected with either the GFP or the GFP-WT vector as above, and 5.0 × 10^6^ cells were used per pull-down. The following antibodies were used: β-tubulin (Developmental Studies Hybridoma Bank E7), Lamin B1 (D4Q4Z, Cell Signaling 12586), E2F4 (4E2F04, Thermo Fisher MA1-26624), GFP (rabbit, Invitrogen A-11122 and mouse, Santa Cruz sc-57587), DP-1 (TFD10, Santa Cruz sc-53642), HCFC1 (Novus Biologicals NB100-68210), YEATS2 (Thermo Fisher PA5-36939), and LIN54 and LIN9 (gift from Dr. Larisa Litovchick). Uncropped images are provided in the Source Data File.

For visualization of GFP in cell lines that express rescue constructs, 0.2 × 10^6^ cells per line were plated onto a gelatinized 35 mm glass bottom dish (Ibidi 81218), and live cell imaging of dishes was performed 24 h later on a Zeiss LSM5 10 Meta Confocal Microscope.

### RT-qPCR and RNA-Seq

All gene expression analyses were performed on cells grown at low density for four days and RNA extraction was performed using the RNeasy micro kit (Qiagen 74004). For RT-qPCR, complementary DNA (cDNA) was synthesized using the ProtoScript First Strand cDNA Synthesis Kit (E6300) and qPCR was performed using the SYBR GreenER Master Mix (Invitrogen) on an ABI 7900HT Detection System, with primers listed in Supplementary Data 13. All samples were run in triplicate and normalized to a *Gapdh* RT-qPCR. For RNA-Seq, libraries were prepared using the TruSeq Stranded mRNA Library Preparation Kit (Illumina RS-122-2101). Four wild-type and four E2F4KO mESC lines were sequenced on a HiSeq4000, generating on average 40 million paired-end (2 × 76) reads per sample. Similarly, two passages of two TKO and two QKO lines were sequenced on a separate run of a HiSeq4000, generating on average 45 million paired-end (2 × 76) reads per sample. Obtained reads were mapped to mouse reference genome mm10 with STAR2.5.1b using default settings with an average mapping rate of 96% (73% to genes). Genes that had at least ten reads in two out of four samples of either the control group or the experimental group were used for further analysis. Differentially expressed genes were obtained using DEseq2.

### ChIP-qPCR and ChIP-Seq

Chromatin immunoprecipitation (ChIP) was done according to a published method^63^. Briefly, mESCs were crosslinked with 1% formaldehyde in PBS for 10 min at room temperature and the reaction was stopped by adding glycine to a final concentration of 1.25 M for 5 min at room temperature. Crosslinked cells were collected with a cell scraper, rinsed twice with PBS, and resuspended at a concentration of 5 × 10^7^ cells/mL of swelling buffer (0.1 M Tris pH 7.6, 10 mM KOAc, 15 mM MgOAc, 1% NP40) to remove the cytoplasmic fraction. Nuclei were resuspended at a concentration of 10 × 10^8^ cells/mL of nuclei lysis buffer (50 mM Tris-Cl pH 8.0, 10 mM EDTA, 1% SDS), and lysate was sonicated for 90 cycles (30 s ON/50 s OFF) in a Diagenode Bioruptor (Diagenode UCD-300). The sonicated chromatin was pre-cleared by adding washed and blocked StaphA at a concentration of 10 µL/1 × 10^7^ cells (Calbiochem 507862). StaphA were washed in dialysis buffer (2 mM EDTA, 50 mM Tris-Cl pH 8.0), and blocked in 10 mg/mL salmon sperm DNA (EMD Millipore 16–157) and 10 mg/mL BSA. For each IP, 100 µL of pre-cleared chromatin was diluted in twice the volume of IP dilution buffer (0.01% SDS, 1.1% Triton X-100, 1.2 mM EDTA, 16.7 mM Tris–HCl pH 8.0, 167 mM NaCl) and 5 µg of antibody was added. Antibodies against E2F4 (C-20, Santa Cruz sc-866X), H3K4me3 (Abcam ab8580) and H3K9ac (Active Motif 39137) were used. IP and 10% input samples were rotated overnight at 4 °C and pull-down was performed with washed/blocked StaphA at a concentration of 10 µL/IP. StaphA pellets were washed twice with dialysis buffer, then four times with IP wash buffer (100 mM Tris–Cl pH 9.0, 500 mM LiCl, 1% NP40, 1% Deoxycholic Acid). IP elution buffer (50 mM NaHCO_3_, 1% SDS) was added and samples were vortexed to release protein/DNA complexes from StaphA. Crosslinks were reversed by incubation in elution buffer + 0.2 M NaCl at 67 °C for 4 h. Sonicated DNA was purified using the Qiagen QIAquick PCR purification kit (Qiagen 28104). ChIP-qPCR was performed using the SYBR GreenER Master Mix (Invitrogen) on an ABI 7900HT Detection System, with primers listed in Supplementary Data 13.

For ChIP-Seq, libraries of two biological replicates (at different passages) of two TKO clones (TKO2 and TKO3 in Fig. 5) and two QKO clones (QKO1 and QKO2 in Fig. 5). ChIP for H3K4me3 and H3K9ac and corresponding input samples were constructed using the NEBNext DNA Sample Prep Master Mix Set for Illumina (NEBNext E6040) and NEBNext Multiplex Oligos for Illumina (NEBNext E7335 and E7500) according to kit instructions. Libraries were quantified using the KAPA Library Quantification Kit with Universal qPCR Master Mix (KAPA Biosystems, KK4824) according to kit instructions, qPCR was performed using the SYBR GreenER Master Mix (Invitrogen) on an ABI 7900HT Detection System, and pooled samples were sequenced on a HiSeq4000, generating on average 23 million single-end reads per sample. Obtained reads were mapped to mouse reference genome mm9 with bowtie2.3.4. Peakcalling was performed with MACS2.1.1 using broadpeaks, merging the two replicates from identical cell lines, and using the merged input samples as control. Differential peaks were determined with the R package DiffBind (https://bioconductor.org/packages/release/bioc/html/DiffBind.html), again merging the two replicates from identical cell lines and using the peaks generated by MACS. Deeptools was used to visualize the data^64^. For enrichment analysis, genes associated with the peaks were identified by mapping the peaks to the nearest transcription start sites (within 5.0 kb upstream and 1.0 kb downstream) using GREAT.

E2F4-bound genes were obtained from publically available .bed files (GSE48666), by peak calling with MACS and remapping the peaks to the nearest transcription start sites (within 1.0 kb upstream and 1.0 kb downstream) using GREAT^65^.

### Tandem affinity purification and mass spectrometry

Tagged mouse E2F4 was overexpressed in TKO mESCs by lentiviral infection. The lentiviral backbone vector (pWPXLd/LAP-C/puro/DEST) was created by inserting a DEST/TEV cleavage site-S tag-PreScission cleavage site-EGFP/puromycin resistance cassette into pWPXLd vector (a gift from Didier Trono, Addgene plasmid #12258). The Gateway entry vector for mouse E2F4 was generated by cloning E2F4 cDNA from the PiggyBac system into pDONR221. Then the N-terminally LAP (EGFP-TEV cleavage site-S tag-PreScission cleavage site)-tagged mouse E2F4 was generated by LR recombination between E2F4 entry vector and pWPXLd/LAP-N/puro/DEST. Lentiviral infection was performed, as described in ref. ^66^. Following infection for 48 h, GFP-positive cells were isolated by fluorescent-activated cell sorting with a BD FACSAria™ instrument.

Tagged human E2F4 was overexpressed in RPE cells using the Flp-In system. Gateway entry vector for human E2F4 was obtained from Life Technologies (clone number IOH23241). Flp-In system compatible N-terminally LAP (EGFP-TEV cleavage site-S tag-PreScission cleavage site)-tagged human E2F4 was generated by LR recombination between the hE2F4 entry vector and pG-LAP6/puro. Flp-In system compatible RPE cells. RPE cells were transfected with 150 ng of LAP-hE2F4 and 850 ng of pOG44, and selection was performed with 10 µg/mL puromycin. Tandem affinity purification and analysis were performed as previously described^66,67^. The mass spectrometry proteomics data have been deposited to the ProteomeXchange Consortium via the PRIDE^68^ partner repository with the dataset identifier PXD008796 and 10.6019/PXD008796.

### Statistical analysis

Statistical significance was assayed with GraphPad Prism software. Data are represented as mean ± SEM. Unless otherwise specified, each control and experimental line was subjected to at least two biological replicates, and the average of the biological replicates was recorded and used in the *t*-test. GO term enrichment analysis of differentially expressed genes was performed with GOrilla^69^ and visualization was performed using REVIGO^70^ to filter out redundant GO terms. The allowed similarity was decreased to the lowest settings to generate the smallest list of GO terms per set of genes. Unpaired *t*-tests were performed with all individual data points from all the biological replicates. *P*-values for overlap in Venn diagrams were obtained using the hypergeometric test.

### Reporting Summary

Further information on research design is available in the Nature Research Reporting Summary linked to this article.

## Supplementary information

Supplementary Information

Description of Additional Supplementary Files

Supplementary Data 1

Supplementary Data 2

Supplementary Data 3

Supplementary Data 4

Supplementary Data 5

Supplementary Data 6

Supplementary Data 7

Supplementary Data 8

Supplementary Data 9

Supplementary Data 10

Supplementary Data 11

Supplementary Data 12

Supplementary Data 13

Reporting Summary

Source data

## Data Availability

All data generated or analyzed during this study are included in this published article and its supplementary information files or are available from the corresponding author upon reasonable request. RNA-seq and ChIP-seq data are available in the ‘GEO repository’ under the accession number GSE109684. Proteomics datasets are available in the ‘EBI repository’ under the accession number PXD008796. Raw data underlying all Figures are provided as a Source Data file. A reporting summary for this Article is available as a Supplementary Information file.
