# Supplementary material for: E2F4 regulates transcriptional activation in mouse embryonic stem cells independently of the RB family
Source: Nat Commun. 2019 Jul 3;10:2939. doi: 10.1038/s41467-019-10901-x (PMC6610666; doi:10.1038/s41467-019-10901-x)
Supplement: Supplementary file 1 — Supplementary Information [file 41467_2019_10901_MOESM1_ESM.pdf]

SUPPLEMENTARY INFORMATION

E2F4 regulates transcriptional activation in mouse embryonic stem cells independently of the RB family

Hsu *et al.*

Figure S1

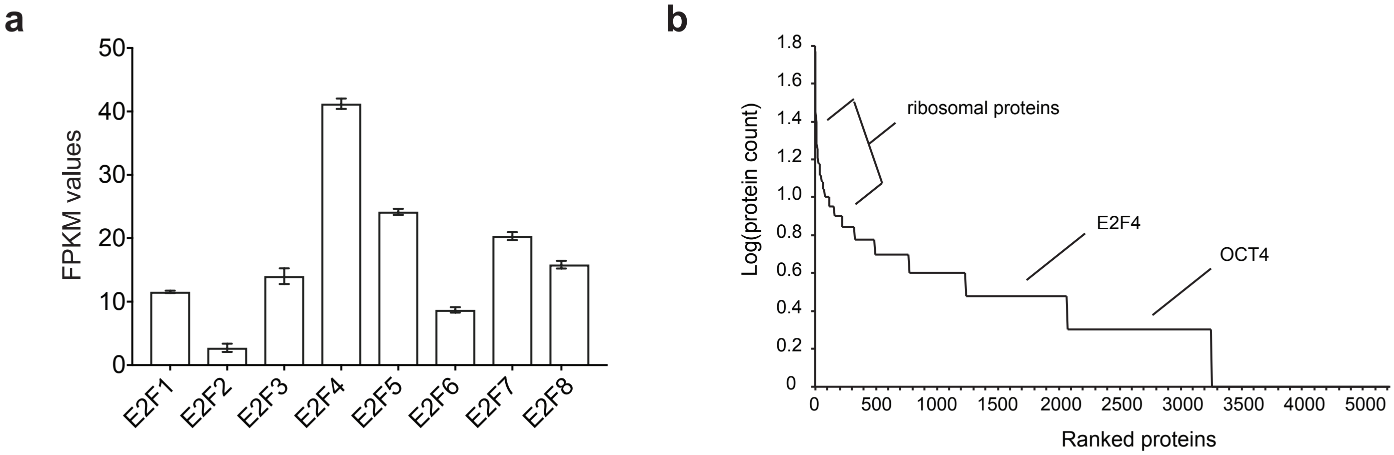

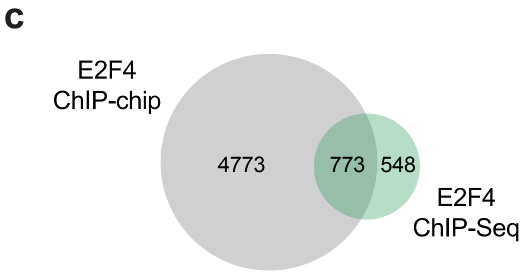

d

Accession #	GO Term	P-value (-log10)
GO:0044237	cellular metabolic process	204.0778
GO:0008152	metabolic process	185.0367
GO:0044260	cellular macromolecule metabolic process	182.3335
GO:0071704	organic substance metabolic process	174.5719
GO:0034641	cellular nitrogen compound metabolic process	173.6271
GO:0044238	primary metabolic process	170.5702
GO:0006807	nitrogen compound metabolic process	170.3757
GO:0043170	macromolecule metabolic process	157.4962
GO:0046483	heterocycle metabolic process	153.1475
GO:0006725	cellular aromatic compound metabolic process	148.0395
GO:1901360	organic cyclic compound metabolic process	145.011
GO:0009987	cellular process	92.3526
GO:0009058	biosynthetic process	92.0306
GO:0044249	cellular biosynthetic process	89.2328
GO:0071840	cellular component organization or biogenesis	83.2154
GO:0006259	DNA metabolic process	68.6162
GO:0022402	cell cycle process	64.6615
GO:0033554	cellular response to stress	61.4377
GO:0007049	cell cycle	57.9666
GO:0006396	RNA processing	54.7932

Supplementary Fig. 1: Expression and DNA-binding activity of E2F4 in mESCs.
a Quantification of the mRNA levels for the E2F-coding genes, based data from RNA-Seq data from two independent biological replicates (Bruce4 mESCs, GSE93453). Data shown as the mean and standard error of the mean. **b** Quantification of E2F4 protein levels based on analysis of the proteome of mESCs (Pfeiffer *et al.*, 2011). E2F4 is the only E2F family member detected in this analysis, indicating that it is the most highly expressed. **c** Overlap of publicly available E2F4 genome-wide ChIP-Seq and proximal promoter ChIP-chip datasets generated in mESCs (Kim *et al.*, 2010). **d** Enrichment of biological processes in the union of the two datasets is shown. GO terms were filtered for redundancy through REVIGO and the top 20 most significant are shown. Source data are provided as a source data file.

Figure S2

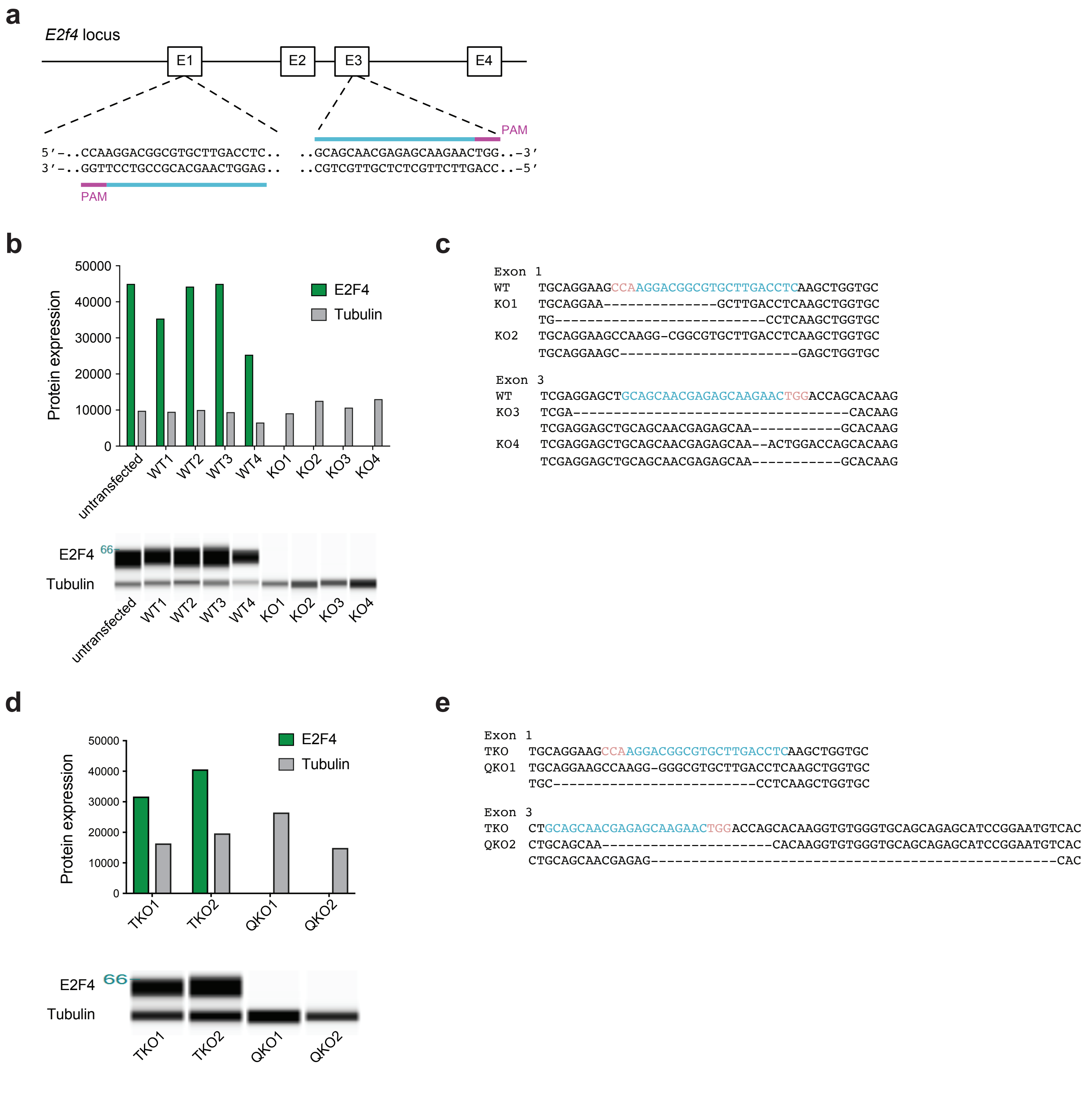

Supplementary Fig. 2: E2F4 knockout in wild-type and RB family TKO mESCs.
a Schematic representation of the mouse *E2f4* locus (4 first exons are shown, not to scale) and the two sgRNAs selected. **b** Quantification of E2F4 protein levels by immunoassay (fluorescence units are shown) in wild-type (WT) and E2F4KO (KO) clones. The trace of the immunoassay (n=1 biological replicate per clone) is shown below the quantification. The 66 kDa marker is shown. **c** Sequence analysis of the *E2f4* locus for four E2F4KO clones (n=4-8 independent PCR products were sequenced for each clone). **d** Quantification of E2F4 protein levels by immunoassay (fluorescence units are shown) in *Rb* family triple knockout (TKO) and quadruple knockout (QKO, TKO with E2F4 knockout) clones (n=1 biological replicate per clone). The trace of the immunoassay is shown below the quantification. **e** Sequence analysis of the *E2f4* locus for two QKO clones (n=4-8 independent PCR products were sequenced for each clone). Source data are provided as a source data file.

Figure S3

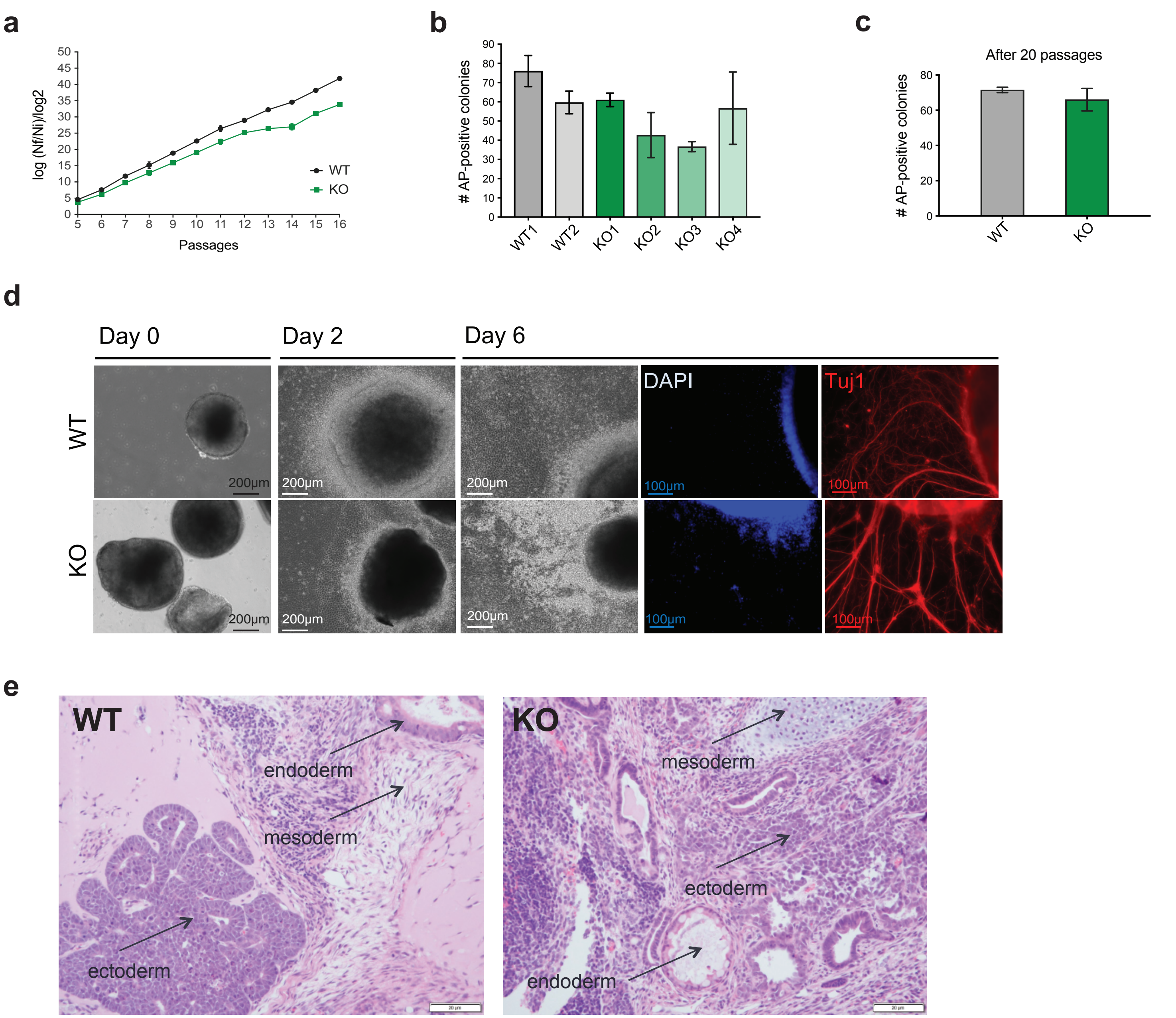

Supplementary Fig. 3: Loss of E2F4 in mESCs does not affect pluripotency and differentiation.
a Long-term proliferation of wild-type (WT) and E2F4KO (KO) mESCs (n=2 clones per genotype; error bars are shorter than the height of the symbol and therefore not shown). Growth rate is depicted as the log ratio of the final cell count (Nf) over the initial number of cells plated (Ni, $3x10^5$ cells) per passage. **b,c** Total number of WT and E2F4KO colonies per well in 6-well plates one week after plating at low density at low passage (b, n=3 biological replicates per clone) and after 20 passages (c, n=2 WT and n=5 KO clones). Colonies were stained for alkaline phosphatase (AP) to count only undifferentiated colonies. Data shown as the mean and standard error of the mean. Differences are not statistically significant. **d** Representative photographs of WT and E2F4KO mESCs undergoing neuronal differentiation induced by retinoic acid treatment. Immunofluorescence at day 6 for the neuronal marker Tuj1 is shown (red). DAPI (blue) marks the DNA (n=2 biological replicates with 2 clones per genotype). Scale bars, 200 μm (brightfield) or 100 μm (fluorescence). **e** Representative photographs of sections from WT and E2F4KO teratomas (n≥4 clones per genotype). Arrows delineate evidence of the three germ layers. Scale bar, 20 μm. Source data are provided as a source data file.

Figure S4

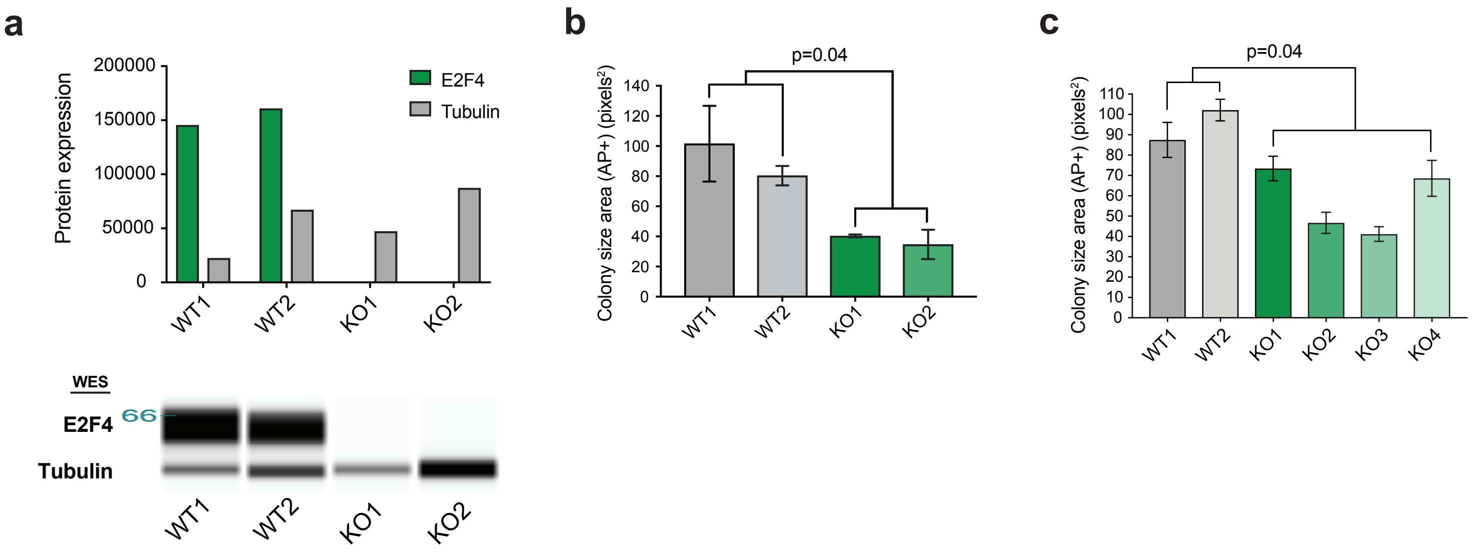

Supplementary Fig. 4: Loss of E2F4 affects the growth of mESCs in different contexts.
a Quantification of E2F4 protein levels by immunoassay (fluorescence units are shown) in wild-type (WT) and E2F4 knockout (KO) clones derived from R1 mESCs. The trace of the immunoassay (n=1 biological replicate per clone) is shown below the quantification. The 66 kDa marker is shown. **b** Quantification of the size of AP^{+} colonies in R1 WT and E2F4KO mESCs (unpaired t-test; n=2 biological replicates per clone). **c** Quantification of the size of AP-positive colonies with mESCs grown in 2i medium (unpaired t-test; n=3 biological replicates per clone). Data shown as the mean and standard error of the mean. Source data are provided as a source data file.

Figure S5

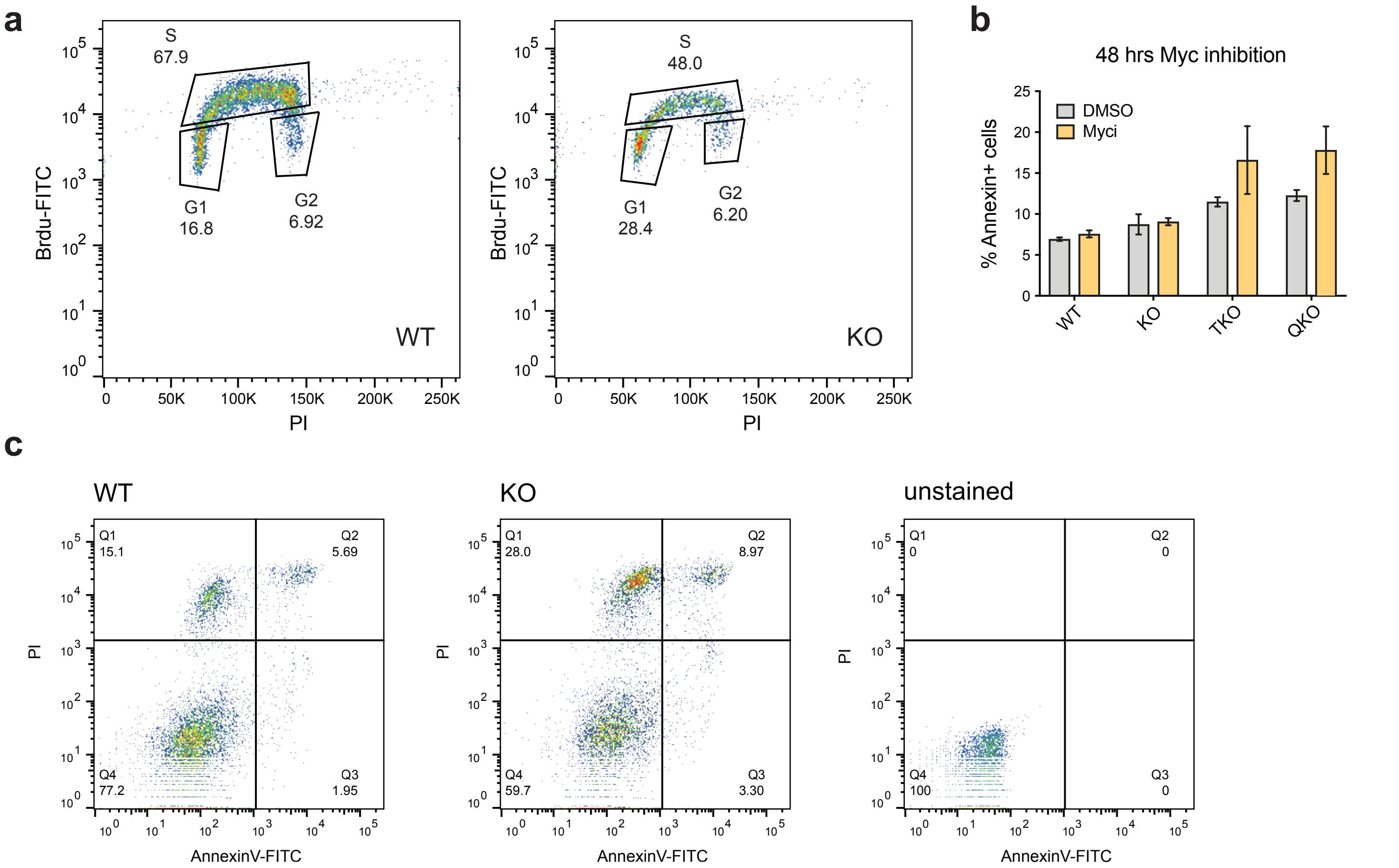

Supplementary Fig. 5: Cell cycle and cell survival analysis of E2F4 KO mESCs.
a Representative FACS plot of BrdU/PI analysis for one wild-type (WT) and one E2F4KO (KO) clone, performed after 4 days of low density plating. **b** Analysis of cell death (Annexin V-positive cells) in mESCs of different genotypes after 48 hours of treatment with a Myc inhibitor (n=1 biological replicate with 3-4 independent clones of each genotype). Data shown as the mean and standard error of the mean. **c** Representative FACS of an Annexin V/PI analysis for one WT and one E2F4KO clone, performed after 4 days of low density plating. Source data are provided as a source data file.

Figure S6

a

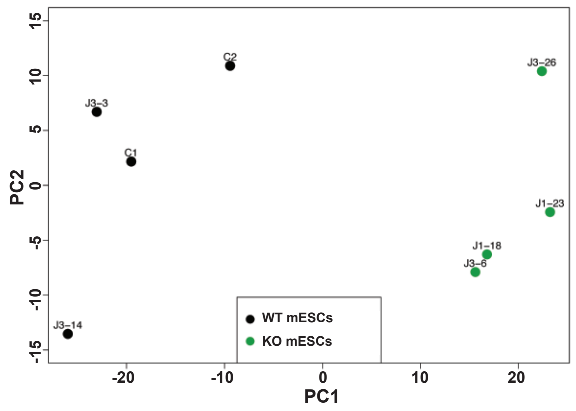

b

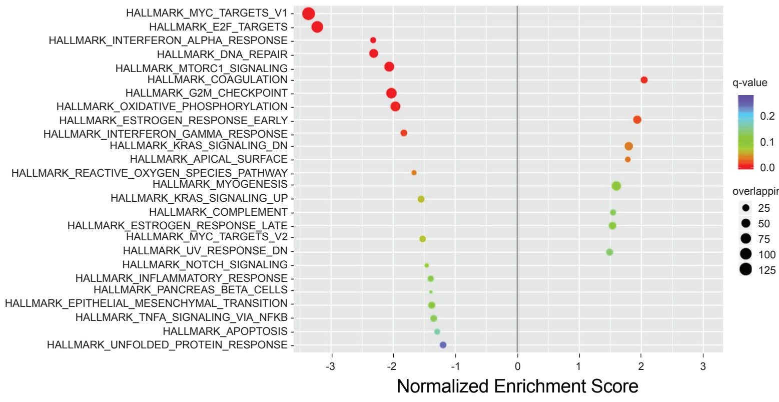

c

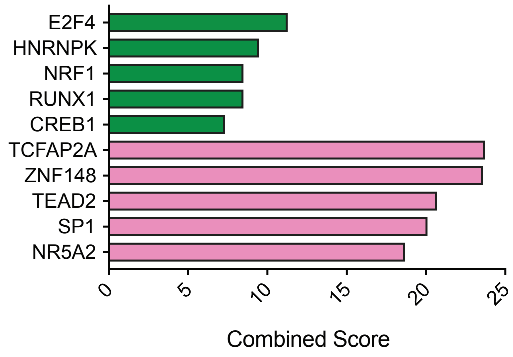

d

Accession #	GO Term	P-value (-log10)
GO:0032501	multicellular organismal process	6.163
GO:0048518	positive regulation of biological process	5.4056
GO:0048522	positive regulation of cellular process	5.1772
GO:0065007	biological regulation	4.684
GO:0032879	regulation of localization	4.6778
GO:0048523	negative regulation of cellular process	4.3215
GO:0007610	behavior	4.3036
GO:0050896	response to stimulus	4.2596
GO:0048519	negative regulation of biological process	4.0595
GO:0065009	regulation of molecular function	3.4584
GO:0009070	serine family amino acid biosynthetic process	3.3036
GO:0001676	long-chain fatty acid metabolic process	3.2741
GO:0001662	behavioral fear response	3.2381
GO:0051049	regulation of transport	3.2083
GO:0032762	mast cell cytokine production	3.1013
GO:1905314	semi-lunar valve development	3.1013
GO:2000810	regulation of bicellular tight junction assembly	3.0635
GO:0051128	regulation of cellular component organization	3.0315

e

Accession #	GO Term	P-value (-log10)
GO:1901342	regulation of vasculature development	12.059
GO:0032502	developmental process	10.8386
GO:0048522	positive regulation of cellular process	9.1798
GO:0048518	positive regulation of biological process	8.2933
GO:0032501	multicellular organismal process	7.9872
GO:0065007	biological regulation	6.1302
GO:0040011	locomotion	6.0097
GO:0040012	regulation of locomotion	5.8416
GO:0002682	regulation of immune system process	5.5229
GO:0035458	cellular response to interferon-beta	5.3635
GO:0042127	regulation of cell proliferation	5.1972
GO:0007156	homophilic cell adhesion via plasma membrane adhesion molecules	5.0391
GO:0006357	regulation of transcription from RNA polymerase II promoter	4.7595
GO:0008283	cell proliferation	4.6234
GO:0022610	biological adhesion	4.51
GO:0048583	regulation of response to stimulus	4.1878
GO:0007610	behavior	4.0747
GO:0048519	negative regulation of biological process	3.7033
GO:0002376	immune system process	3.5834
GO:0006928	movement of cell or subcellular component	3.2976

Accession #	GO Term	P-value (-log10)
GO:0022414	reproductive process	7.2774
GO:0003008	system process	7.1007
GO:0007635	chemosensory behavior	5.466
GO:0032501	multicellular organismal process	4.9172
GO:0006631	fatty acid metabolic process	3.7799
GO:0006690	icosanoid metabolic process	3.7235
GO:0030001	metal ion transport	3.6968
GO:1901568	fatty acid deriv. metab. process	3.5544
GO:0051349	positive regulation of lyase activity	3.3947
GO:0015849	organic acid transport	3.3565
GO:0007186	GPCR signaling pathway	3.1308

Supplementary Fig. 6: Analyses of RNA-Seq data comparing WT and E2F4KO mESCs.
a Principal component analysis (PCA) for wild-type (WT) and E2F4KO (KO) mESCs based on RNA-seq data. **b** GSEA analysis of differentially expressed genes (q-value < 0.05) in E2F4KO mESCs with respect to WT. The MSigDB hallmarks dataset was used. **c** Enrichr analysis of TRANSFAC motifs enriched in genes that are downregulated (green) and upregulated (pink) in E2F4KO mESCs with respect to WT. **d** Biological processes enriched in genes upregulated in E2F4KO mESCs and bound by E2F4 in ChIP data (q-value < 0.05, fold change > 0.5 (log2)). **e** Biological processes enriched in genes downregulated (left) and upregulated (right) in both E2F4KO and QKO mESCs compared to WT and TKO, respectively. GO terms were filtered for redundancy through REVIGO and the top 20 most significant are shown.

Figure S7

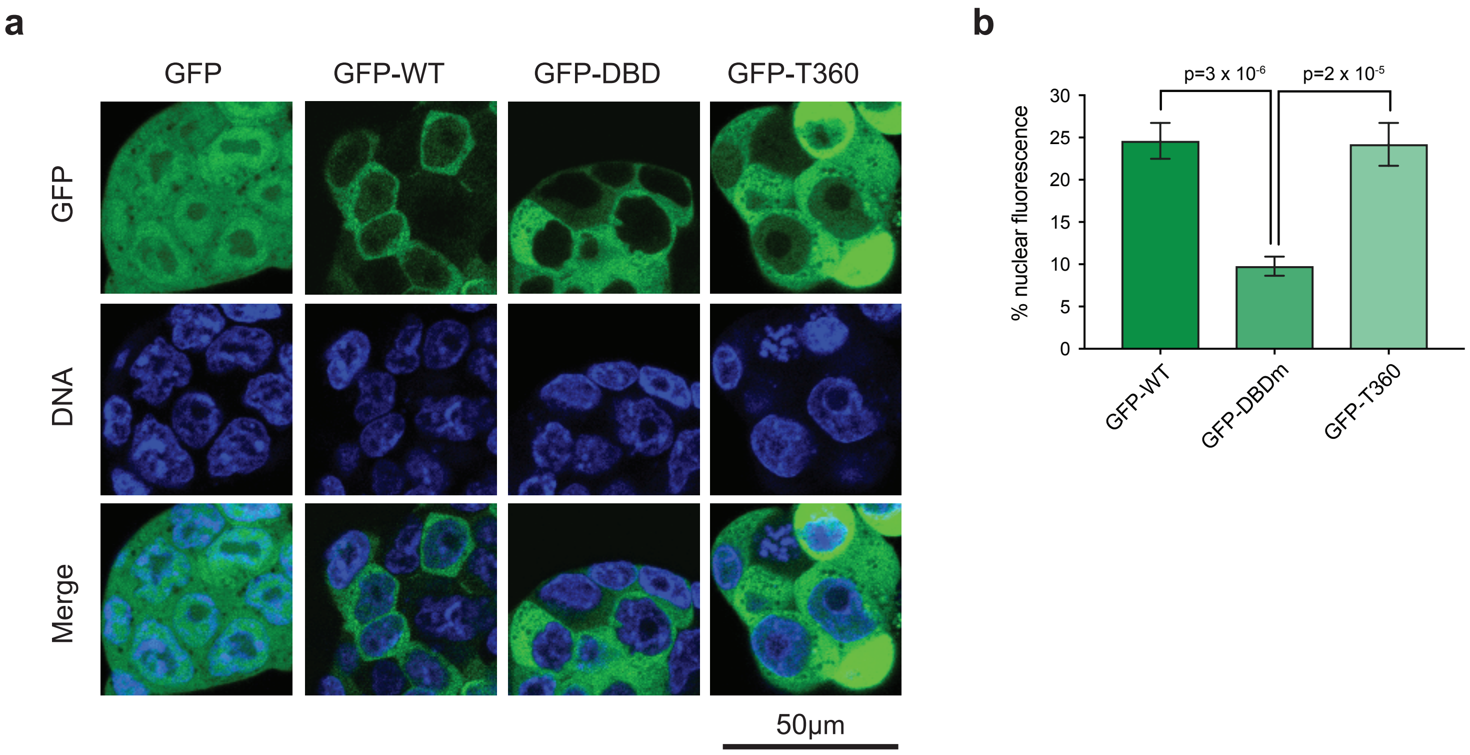

Supplementary Fig. 7: Expression of rescue constructs.
a Live-cell imaging of wild-type cells transfected with the rescue constructs (GFP^+) and stained with Hoechst (blue). Scale bar, 50 µm. **b** Quantification of nuclear fluorescence as a readout of the nuclear localization of each construct. Nuclear fluorescence is graphed as a percentage of total cell fluorescence. An unpaired t-test was performed using all data points from 6-13 cells per condition. Data shown as the mean and standard error of the mean. Source data are provided as a source data file.

Figure S8

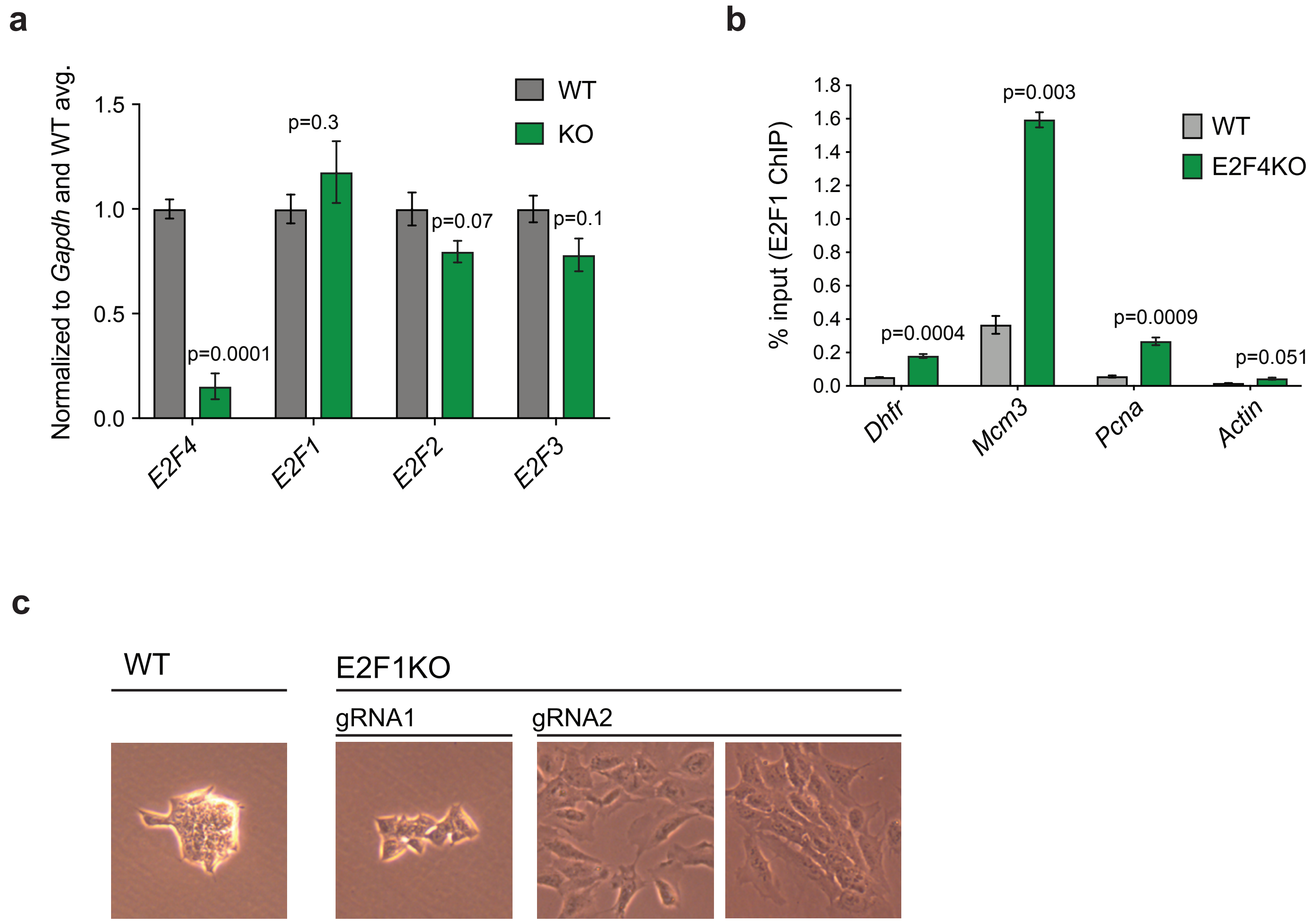

Supplementary Fig. 8: E2F1 can compensate partially for loss of E2F4.
a RT-qPCR validation of canonical activator E2Fs. Gene expression in WT and E2F4KO (KO) mESCs for the genes indicated was normalized to *Gapdh* expression and then to expression levels in WT cells (unpaired t-test; n=1 biological replicate with 3 WT and 3-4 E2F4KO clones). **b** Quantification of E2F1 binding to target genes and an *Actin* negative control in WT and E2F4KO mESCs (unpaired t-test; n=2-3 biological replicates with 1 WT and 1 E2F4KO clone). Binding was normalized to 10% input. Data shown as the mean and standard error of the mean. Source data are provided as a source data file. **c** Representative brightfield images of wild-type (WT) and E2F1KO colonies (same magnification). Cells were selected for transfection of the E2F1 gRNAs or empty vector control, passaged twice, and then plated as single cells at 1000 cells/mL. Images were taken 3 days after plating single cells.

Figure S9

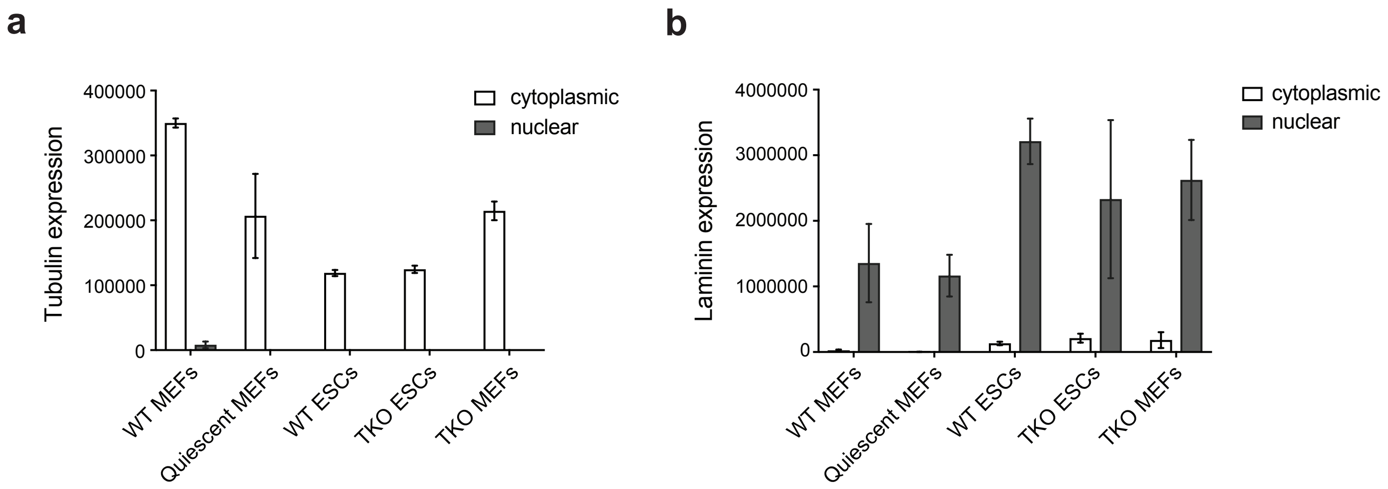

Supplementary Fig. 9: Analysis of cellular fractionation of WT and TKO cells.
a,b Quantification of cytoplasmic and nuclear Tubulin (a) and Laminin (b) expression by immunoassay (direct fluorescence units are shown) in cycling and quiescent mouse embryonic fibroblasts (MEFs), WT and TKO mESCs, and TKO MEFs (n=2-3 biological replicates per cell type). Data shown as the mean and standard error of the mean. Source data are provided as a source data file.

Figure S10

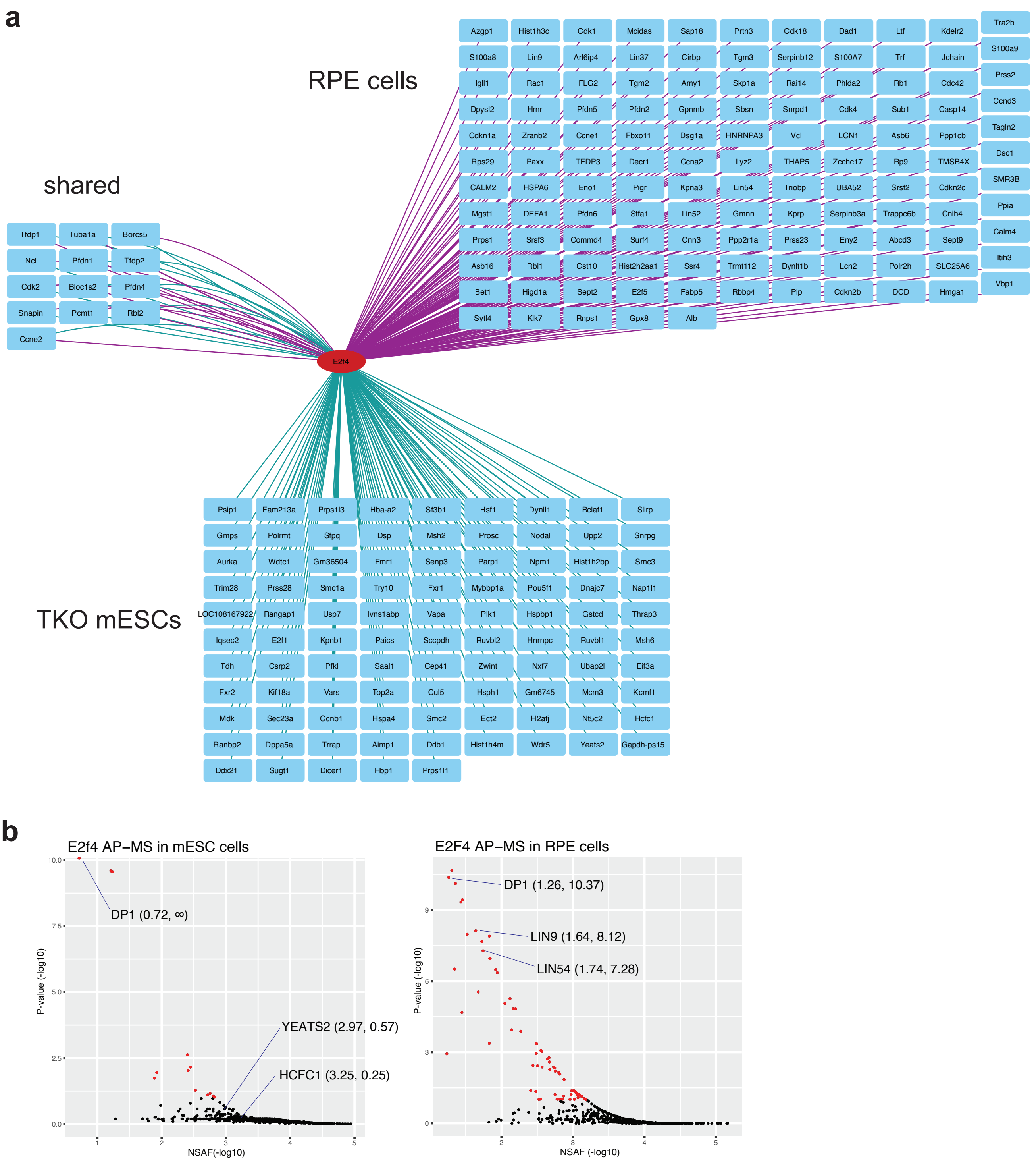

Supplementary Fig. 10: Identification of the E2F4 interactome.
a Candidate interactors of E2F4 in retinal pigment epithelium (RPE) cells, mESCs, and shared between the two cell types. Purple and teal lines represent interactions in RPE cells and mESCs, respectively. **b** Volcano plot of candidate E2F4 interactors identified in mESCs (left) and RPE cells (right) by AP-MS. x-axis represents -log10 tranformation of normalized spectral abundance factor (NSAF), and y-axis represents -log10 transformation of p-value. Locations of DP1, YEATS2, HCFC1, LIN9, and LIN54 are indicated.

Figure S11

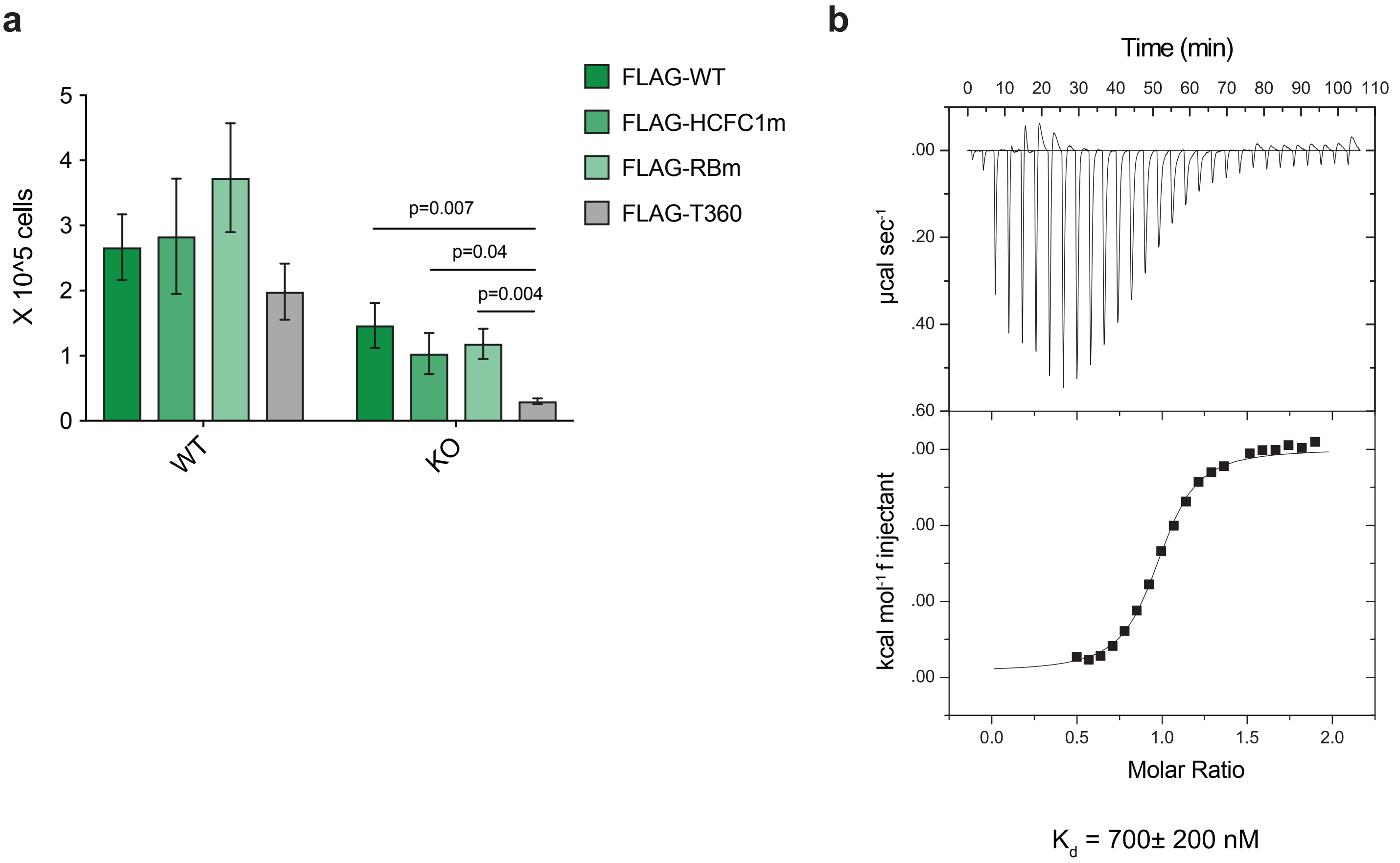

Supplementary Fig. 11: Validation of the E2F4 interactome in cells and *in vitro*.
a Total number of cells per well in 6-well plates one week after plating transfected cells at low density (unpaired t-test was performed with all individual data points from n=2-4 biological replicates with 2 WT and 2 E2F4KO clones in each replicate). The T360 truncation mutant was used as a control unable to rescue the consequences of E2F4 loss (as in Figure 4d). Data shown as the mean and standard error of the mean. **b** Isothermal titration calorimetry data for purified E2F4 transactivation domain binding to the CBP TAZ1 domain. The reported Kd value is the average from three replicates of the shown experiment with the standard deviation reported as error. Source data are provided as a source data file.

Figure S12

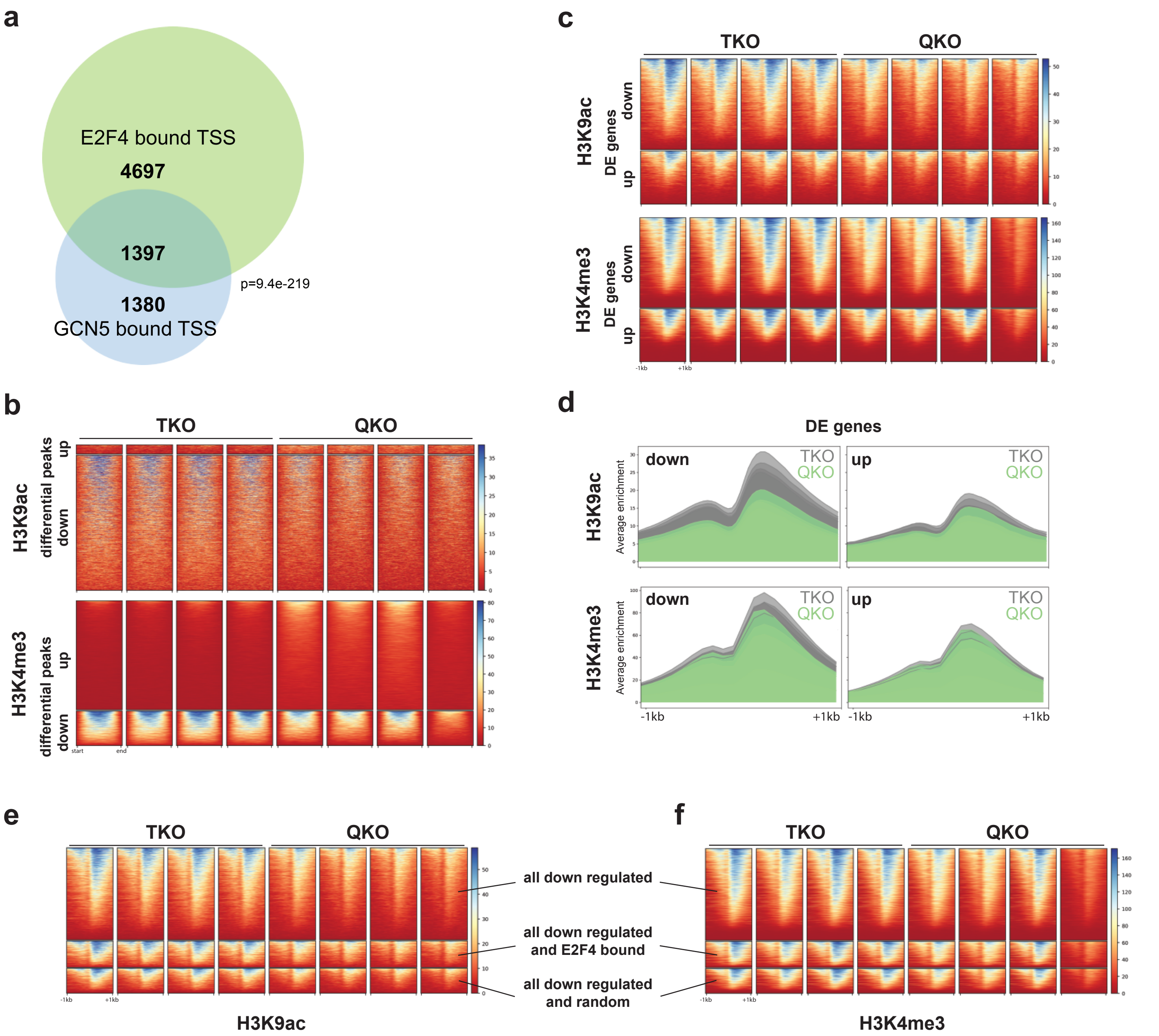

Supplementary Fig. 12: ChIP-seq analysis of E2F4 role in gene activation.
a Analysis of the overlap between E2F4 and GCN5 binding at transcription start sites (TSS) from published datasets in mouse ES cells. E2F4 data are from a merged dataset of ChIP-chip and ChIP-seq, as listed in Supplementary Table S4. GCN5 data are from Hirsch et al. (see text). **b** Heatmap of enrichment scores of H3K9ac and H3K4me3 ChIP-seq signal across differential peaks (all peaks from beginning to end are shown as the same size). **c** Heatmap of enrichment scores of H3K9ac and H3K4me3 ChIP-seq signal across differentially expressed genes (up-regulated and down-regulated in QKO mESCs compared to TKO mESCs) (-1/+1kb). **d** Average enrichment of H3K9ac and H3K4me3 signal around TSS (-1/+1kb) of up- and downregulated genes. **e,f** Heatmap of enrichment scores of H3K9ac (e) and H3K4me3 (f) signal around TSS (-1/+1kb) associated with downregulated genes in QKO cells compared to TKO cells, genes that are downregulated and bound by E2F4, and a random set of downregulated genes.

Figure S13

a

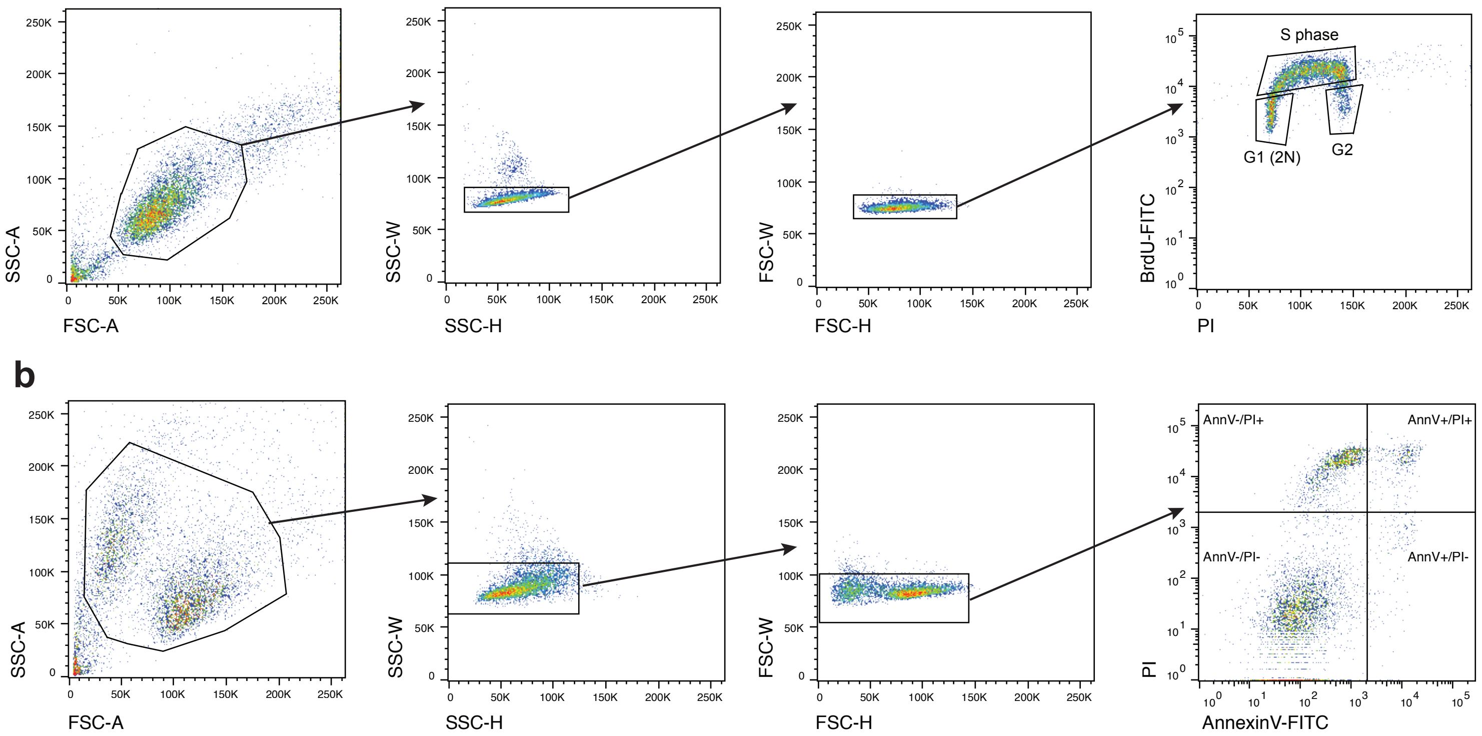

Supplementary Fig. 13: Gating strategies used for cell sorting.
a Gating strategy to quantify the % of cells in G1, S, and G2 phases for experiments presented in Fig. 2a and 5d, as well as the % of cells with 2N DNA content (2N) and in S phase for experiments presented in Fig. 2b and 2c. **b** Gating strategy to quantify cell viability (% AnnexinVneg/PIneg) for experiments presented in Fig. 2d and 5e, and the rate of cell death (% AnnexinVpos) for experiment presented in Supplementary Fig. 5b.
