# Supplementary material for: E2F4 regulates transcriptional activation in mouse embryonic stem cells independently of the RB family
Source: Nat Commun. 2019 Jul 3;10:2939. doi: 10.1038/s41467-019-10901-x (PMC6610666; doi:10.1038/s41467-019-10901-x)
Supplement: Supplementary file 2 — Description of Additional Supplementary Files [file 41467_2019_10901_MOESM2_ESM.pdf]

Description of Additional Supplementary Files

File name: Supplementary Data 1

Description: RNA-seq analysis of WT and E2F4KO mESCs. Number of reads and fragments per million (FPM) mapped fragments are shown, with p-values and adjusted p-values.

File name: Supplementary Data 2

Description: Enrichment of TRANSFAC motifs in the promoter regions of genes downregulated in E2F4KO mESCs relative to WT. Genes associated with each term and statistical analyses are shown.

File name: Supplementary Data 3

Description: Enrichment of TRANSFAC motifs in the promoter regions of genes upregulated in E2F4KO mESCs relative to WT. Genes associated with each term and statistical analyses are shown.

File name: Supplementary Data 4

Description: Integration of ChIP and RNA-seq datasets to identify direct targets of E2F4 in mESCs.

File name: Supplementary Data 5

Description: RNA-seq analysis of TKO (Rb family triple knockout) and QKO (TKO with the additional E2F4KO) mESCs. Number of reads and fragments per million (FPM) mapped fragments are shown, with p-values and adjusted p-values.

File name: Supplementary Data 6

Description: Candidate cofactors of E2F4 as identified by affinity-purification mass spectrometry (AP-MS).

File name: Supplementary Data 7

Description: Interactions between E2F4 and DP family proteins in RPE cells and in ES cells from affinity-purification mass spectrometry (AP-MS).

File name: Supplementary Data 8

Description: Post-translational modifications of E2F4 as identified by affinity-purification mass spectrometry (AP-MS) in human RPE cells and in mouse ES cells. Mod: modification.

File name: Supplementary Data 9

Description: H3K9ac ChIP-seq peaks upregulated in QKO mESCs compared to TKO mESCs. The location of the detected peaks and the statistical analyses are shown.

File name: Supplementary Data 10

Description: H3K9ac ChIP-seq peaks downregulated in QKO mESCs compared to TKO mESCs. The location of the detected peaks and the statistical analyses are shown.

File name: Supplementary Data 11

Description: H3K4me3 ChIP-seq peaks upregulated in QKO mESCs compared to TKO mESCs. The location of the detected peaks and the statistical analyses are shown.

File name: Supplementary Data 12

Description: H3K4me3 ChIP-seq peaks downregulated in QKO mESCs compared to TKO mESCs. The location of the detected peaks and the statistical analyses are shown.

File name: Supplementary Data 13

Description: List and sequence (5’ to 3’) of the primers for RT-qPCR and ChIP-qPCR analysis.
