# Supplementary material for: E2F4 regulates transcriptional activation in mouse embryonic stem cells independently of the RB family
Source: Nat Commun. 2019 Jul 3;10:2939. doi: 10.1038/s41467-019-10901-x (PMC6610666; doi:10.1038/s41467-019-10901-x)
Supplement: Supplementary file 16 — Reporting Summary [file 41467_2019_10901_MOESM16_ESM.pdf]

nature research

Corresponding author(s): Julien Sage

Last updated by author(s): May 10, 2019

Reporting Summary

Nature Research wishes to improve the reproducibility of the work that we publish. This form provides structure for consistency and transparency in reporting. For further information on Nature Research policies, see Authors & Referees and the Editorial Policy Checklist.

Statistics

For all statistical analyses, confirm that the following items are present in the figure legend, table legend, main text, or Methods section.

n/a	Confirmed	
☐	☒	The exact sample size (*n*) for each experimental group/condition, given as a discrete number and unit of measurement
☐	☒	A statement on whether measurements were taken from distinct samples or whether the same sample was measured repeatedly
☐	☒	The statistical test(s) used AND whether they are one- or two-sided<br>*Only common tests should be described solely by name; describe more complex techniques in the Methods section.*
☒	☐	A description of all covariates tested
☐	☒	A description of any assumptions or corrections, such as tests of normality and adjustment for multiple comparisons
☐	☒	A full description of the statistical parameters including central tendency (e.g. means) or other basic estimates (e.g. regression coefficient) AND variation (e.g. standard deviation) or associated estimates of uncertainty (e.g. confidence intervals)
☐	☒	For null hypothesis testing, the test statistic (e.g. *F*, *t*, *r*) with confidence intervals, effect sizes, degrees of freedom and *P* value noted<br>*Give P values as exact values whenever suitable.*
☒	☐	For Bayesian analysis, information on the choice of priors and Markov chain Monte Carlo settings
☐	☒	For hierarchical and complex designs, identification of the appropriate level for tests and full reporting of outcomes
☐	☒	Estimates of effect sizes (e.g. Cohen's *d*, Pearson's *r*), indicating how they were calculated

Our web collection on statistics for biologists contains articles on many of the points above.

Software and code

Policy information about availability of computer code

Data collection	RNA-seq: STAR2.5.1b<br>ChIP-seq: bowtie2.3.4 - MACS2.1.1<br>mass spectrometry: Thermo Fisher Orbitrap Velos/Fusion software
Data analysis	Statistical significance was assayed with GraphPad Prism 7.04 software. For differential analysis and visualization of ChIP-seq data, DiffBind (https://bioconductor.org/packages/release/bioc/html/DiffBind.html) and DeepTools3 (http://deeptools.ie-freiburg.mpg.de/) was used. Genes associated with the peaks were identified by mapping the peaks to the nearest transcription start sites (within 5.0 kb upstream and 1.0 kb downstream) using GREAT 3.0.0 (http://great.stanford.edu/public/html/). For differential analysis of RNA-seq data, DEseq2 was used. GO term enrichment analysis of differentially expressed genes was performed with GOrilla (http://cbl-gorilla.cs.technion.ac.il/) and visualization was performed using REVIGO (http://revigo.irb.hr/).<br>Mass spec Thermo raw files were analyzed using Byonic (Protein Metrics, Inc) software to identify peptides/proteins and were further processed by in-house (lab of Peter Jackson) python and R scripts.

For manuscripts utilizing custom algorithms or software that are central to the research but not yet described in published literature, software must be made available to editors/reviewers. We strongly encourage code deposition in a community repository (e.g. GitHub). See the Nature Research guidelines for submitting code & software for further information.

Data

Policy information about availability of data

All manuscripts must include a data availability statement. This statement should provide the following information, where applicable:

- Accession codes, unique identifiers, or web links for publicly available datasets
- A list of figures that have associated raw data
- A description of any restrictions on data availability

All data generated or analysed during this study are included in this published article (and its supplementary information files) and/or are available in the GEO

repository (for the RNA-seq and ChIP-seq data, https://www.ncbi.nlm.nih.gov/geo/query/acc.cgi?acc=GSE109684) and in the EBI repository (for the proteomics datasets, https://www.ebi.ac.uk/pride/archive/projects/PXD008796) or are available from the authors upon request.

Field-specific reporting

Please select the one below that is the best fit for your research. If you are not sure, read the appropriate sections before making your selection.

☒ Life sciences ☐ Behavioural & social sciences ☐ Ecological, evolutionary & environmental sciences

For a reference copy of the document with all sections, see nature.com/documents/nr-reporting-summary-flat.pdf

Life sciences study design

All studies must disclose on these points even when the disclosure is negative.

Sample size	No statistical methods were used to determine the sample sizes. Our sample sizes are similar to those generally employed in the field and as extensively used in our previously published studies. The exact n values for each experiment can be found in the figure legends or the method section.
Data exclusions	No data were excluded.
Replication	All replication attempts were successful.
Randomization	Randomization of samples in groups was not relevant to this study.
Blinding	The investigator was blinded for measuring colony sizes in one of the low density plating assays for WT and E2F4 KO mESCs. No further blinding for cell culture experiments was performed.

Reporting for specific materials, systems and methods

We require information from authors about some types of materials, experimental systems and methods used in many studies. Here, indicate whether each material, system or method listed is relevant to your study. If you are not sure if a list item applies to your research, read the appropriate section before selecting a response.

Materials & experimental systems

n/a	Involved in the study	
☐	☒	Antibodies
☐	☒	Eukaryotic cell lines
☒	☐	Palaeontology
☐	☒	Animals and other organisms
☒	☐	Human research participants
☒	☐	Clinical data

Methods

n/a	Involved in the study	
☐	☒	ChIP-seq
☐	☒	Flow cytometry
☒	☐	MRI-based neuroimaging

Antibodies

Antibodies used	β-tubulin (Developmental Studies Hybridoma Bank E7)<br>Lamin B1 (D4Q4Z, Cell Signaling 12586)<br>E2F4 (4E2F04, Thermo Fisher MA1-26624) (for immunoblot/assay)<br>GFP (rabbit, Invitrogen A-11122 and mouse, Santa Cruz sc-57587)<br>DP-1 (TFD10, Santa Cruz sc-53642)<br>HCFC1 (Novus Biologicals NB100-68210)<br>YEATS2 (Thermo Fisher PA5-36939)<br>LIN54 and LIN9 antibodies were gift from Dr. Larisa Litovchick (described in Litovchick et al., Mol Cell 2007)<br>Tuj1 antibody (BioLegend 802001)<br>E2F4 (C-20, Santa Cruz sc-866X) (for ChIP)<br>H3K4me3 (Abcam ab8580)<br>H3K9Ac (Active Motif 39137)
Validation	We relied on previous publications, as well as the size of the detected signal in immunoblots and immunoassays.

Eukaryotic cell lines

Policy information about cell lines

Cell line source(s)	ES cell lines J1 and R1 were obtained from the laboratory of Dr. Rudolf Jaenish, MIT. MEFs were generated in the Sage lab. TKO mESCs were generated in the Sage lab and the te Riele lab. RPE cells were obtained from ATCC.
Authentication	None of these ES cell lines were further authenticated. MEFs were genotyped.
Mycoplasma contamination	All the cell lines used were tested on a regular basis for mycoplasma contamination and were always negative in all the experiments performed.
Commonly misidentified lines (See ICLAC register)	Not applicable.

Animals and other organisms

Policy information about studies involving animals; ARRIVE guidelines recommended for reporting animal research

Laboratory animals	NOD scid gamma mice were pruchased from the Jackson Laboratory.
Wild animals	Not applicable.
Field-collected samples	Not applicable.
Ethics oversight	All animal studies were approved by the Administrative Panel on Laboratory Animal Care at Stanford University. All relevant ethical regulations for animal testing and research were complied with. Protocols were approved by the Stanford IACUC.

Note that full information on the approval of the study protocol must also be provided in the manuscript.

ChIP-seq

Data deposition

☒ Confirm that both raw and final processed data have been deposited in a public database such as GEO.

☐ Confirm that you have deposited or provided access to graph files (e.g. BED files) for the called peaks.

Data access links<br>*May remain private before publication.*	https://www.ncbi.nlm.nih.gov/geo/query/acc.cgi?acc=GSE109684
Files in database submission	GSM3500766 D1-10_input_Rep1<br>GSM3500767 D1-10_input_Rep2<br>GSM3500768 D1-10_H3K4me3-ChIP_Rb-TKO_Rep1<br>GSM3500769 D1-10_H3K4me3-ChIP_Rb-TKO_Rep2<br>GSM3500770 D1-10_H3K9ac-ChIP_Rb-TKO_Rep1<br>GSM3500771 D1-10_H3K9ac-ChIP_Rb-TKO_Rep2<br>GSM3500772 D1-2_input_D1-2-Rep1<br>GSM3500773 D1-2_input_D1-2-Rep2<br>GSM3500774 D1-2_H3K4me3-ChIP_Rep1<br>GSM3500775 D1-2_H3K4me3-ChIP_Rep2<br>GSM3500776 D1-2_H3K9ac-ChIP_Rep1<br>GSM3500777 D1-2_H3K9ac-ChIP_Rep2<br>GSM3500778 D1-9_input_Rep1<br>GSM3500779 D1-9_input_Rep2<br>GSM3500780 D1-9_H3K4me3-ChIP_Rep1<br>GSM3500781 D1-9_H3K4me3-ChIP_Rep2<br>GSM3500782 D1-9_H3K9ac-ChIP_Rep1<br>GSM3500783 D1-9_H3K9ac-ChIP_Rep2<br>GSM3500784 D3-8_input_Rep1<br>GSM3500785 D3-8_input_Rep2<br>GSM3500786 D3-8_H3K4me3-ChIP_Rep1<br>GSM3500787 D3-8_H3K4me3-ChIP_Rep2<br>GSM3500788 D3-8_H3K9ac-ChIP_Rep1<br>GSM3500789 D3-8_H3K9ac-ChIP_Rep2<br>GSE109684_Differentialpeaks.xlsx
Genome browser session (e.g. UCSC)	no longer applicable

Methodology

Replicates	Two biological replicates (at different passages) of two TKO clones (TKO2 and TKO3 in Fig. 5) and two QKO clones (QKO1 and QKO2 in Fig. 5) and corresponding input samples
Sequencing depth	D1-10-10-percent-Rep-1 - total reads: 25795828 - uniquely mapped reads: 17250623 - single end - 75 bp<br>D1-10-10-percent-Rep-2 - total reads: 20558992 - uniquely mapped reads: 13696513 - single end - 75 bp<br>D1-10-H3K4me3-Rep-1 - total reads: 21919744 - uniquely mapped reads: 16324524 - single end - 75 bp<br>D1-10-H3K4me3-Rep-2 - total reads: 20131903 - uniquely mapped reads: 14726227 - single end - 75 bp<br>D1-10-H3K9ac-Rep-1 - total reads: 24449814 - uniquely mapped reads: 18865934 - single end - 75 bp<br>D1-10-H3K9ac-Rep-2 - total reads: 21748703 - uniquely mapped reads: 14698468 - single end - 75 bp<br>D1-2-10-percent-Rep-1 - total reads: 34115630 - uniquely mapped reads: 22908837 - single end - 75 bp<br>D1-2-10-percent-Rep-2 - total reads: 22159504 - uniquely mapped reads: 14844310 - single end - 75 bp<br>D1-2-H3K4me3-Rep-1 - total reads: 27678491 - uniquely mapped reads: 19877099 - single end - 75 bp<br>D1-2-H3K4me3-Rep-2 - total reads: 21116751 - uniquely mapped reads: 15019818 - single end - 75 bp<br>D1-2-H3K9ac-Rep-1 - total reads: 23276854 - uniquely mapped reads: 18102617 - single end - 75 bp<br>D1-2-H3K9ac-Rep-2 - total reads: 20710114 - uniquely mapped reads: 14448422 - single end - 75 bp<br>D1-9-10-percent-Rep-1 - total reads: 24738545 - uniquely mapped reads: 16386422 - single end - 75 bp<br>D1-9-10-percent-Rep-2 - total reads: 22420055 - uniquely mapped reads: 14964364 - single end - 75 bp<br>D1-9-H3K4me3-Rep-1 - total reads: 25469692 - uniquely mapped reads: 18612380 - single end - 75 bp<br>D1-9-H3K4me3-Rep-2 - total reads: 20157269 - uniquely mapped reads: 13937583 - single end - 75 bp<br>D1-9-H3K9ac-Rep-1 - total reads: 26708766 - uniquely mapped reads: 19513804 - single end - 75 bp<br>D1-9-H3K9ac-Rep-2 - total reads: 21097040 - uniquely mapped reads: 14208891 - single end - 75 bp<br>D3-8-10-percent-Rep-1 - total reads: 24959236 - uniquely mapped reads: 16471742 - single end - 75 bp<br>D3-8-10-percent-Rep-2 - total reads: 18595401 - uniquely mapped reads: 12470132 - single end - 75 bp<br>D3-8-H3K4me3-Rep-1 - total reads: 20748963 - uniquely mapped reads: 14641895 - single end - 75 bp<br>D3-8-H3K4me3-Rep-2 - total reads: 20184300 - uniquely mapped reads: 13607510 - single end - 75 bp<br>D3-8-H3K9ac-Rep-1 - total reads: 21421495 - uniquely mapped reads: 15876194 - single end - 75 bp<br>D3-8-H3K9ac-Rep-2 - total reads: 20702911 - uniquely mapped reads: 13175907 - single end - 75 bp
Antibodies	H3K4me3 (Abcam ab8580)<br>H3K9Ac (Active Motif 39137)
Peak calling parameters	Obtained reads were mapped to mouse reference genome mm9 with bowtie2.3.4. Peakcalling was performed with MACS2.1.1 using broadpeaks , merging the two replicas from identical cell lines, and using the merged input samples as control.
Data quality	FastQC was used to check sequencing quality.<br>D1-2H3K4me3 - total broad peaks (q-value < 0.01): 74917 - peaks with enrichment > 5fold: 31365<br>D1-10H3K4me3 - total broad peaks (q-value < 0.01): 77019 - peaks with enrichment > 5fold: 31625<br>D1-9H3K4me3 - total broad peaks (q-value < 0.01): 92198 - peaks with enrichment > 5fold: 33394<br>D3-8H3K4me3 - total broad peaks (q-value < 0.01): 83892 - peaks with enrichment > 5fold: 27538<br>D1-2H3K9ac - total broad peaks (q-value < 0.01): 100064 - peaks with enrichment > 5fold: 12780<br>D1-10H3K9ac - total broad peaks (q-value < 0.01): 104218 - peaks with enrichment > 5fold: 10568<br>D1-9H3K9ac - total broad peaks (q-value < 0.01): 97041 - peaks with enrichment > 5fold: 8246<br>D3-8H3K9ac - total broad peaks (q-value < 0.01): 88672 - peaks with enrichment > 5fold: 6583
Software	Differential peaks were determined with the R package DiffBind (https://bioconductor.org/packages/release/bioc/html/DiffBind.html), merging the two replicas from the identical cell line and using the peaks generated by MACS. Deeptools3 was used to visualize the data (http://deeptools.ie-freiburg.mpg.de/). For enrichment analysis, genes associated with the peaks were identified by mapping the peaks to the nearest transcription start sites (within 5.0 kb upstream and 1.0 kb downstream) using GREAT version 3.0.0 (http://great.stanford.edu/public/html/)

Flow Cytometry

Plots

Confirm that:

☒ The axis labels state the marker and fluorochrome used (e.g. CD4-FITC).

☒ The axis scales are clearly visible. Include numbers along axes only for bottom left plot of group (a 'group' is an analysis of identical markers).

☒ All plots are contour plots with outliers or pseudocolor plots.

☒ A numerical value for number of cells or percentage (with statistics) is provided.

Methodology

Sample preparation	For analysis of cell cycle structure, mESCs cells were plated at low density for 4 days and pulsed with BrdU for 3 hours prior to trypsinization, before BrdU and propidium iodide (PI) staining and analysis. Quantification of cell death was performed using AnnexinV-FITC and PI according to the manufacturer's instructions (BD Biosciences).

Instrument	BD FACSAria™
Software	Flowjo was used the the analysis.
Cell population abundance	No cell sorting was performed.
Gating strategy	The gating strategy is shown in Supplementary Figure 13, and the boundaries for "positive" and "negative" cells are indicated based on control antibodies.

☒ Tick this box to confirm that a figure exemplifying the gating strategy is provided in the Supplementary Information.
